# The ZFP36 Family as a Post-Transcriptional Immune Checkpoint in Immunity and Disease: Molecular Mechanisms and Functional Implications

**DOI:** 10.3390/biom16071023

**Published:** 2026-07-13

**Authors:** Yuting Yang, Wenhao Zhong, Qiang Huang, Zichang Liu, Yanwei Wu, Lingjie Luo, Liang Chen

**Affiliations:** 1 School of Medicine, Shanghai University, Baoshan Campus, Shanghai 200444, Chinawuyanwei@shu.edu.cn (Y.W.); 2 Institute of Artificial Intelligence and Biomanufacturing, Shanghai University, Shanghai 200444, China; 3 Xinhua Hospital, Shanghai Jiao Tong University School of Medicine, Shanghai 200092, China

**Keywords:** ZFP36 family, TTP, RNA-binding proteins, AU-rich elements, post-transcriptional regulation, immune homeostasis

## Abstract

The zinc finger protein 36 (ZFP36) family, including ZFP36/tristetraprolin (TTP), ZFP36 CCCH-type-like 1 (ZFP36L1), and ZFP36 CCCH-type-like 2 (ZFP36L2), consists of conserved CCCH-type tandem zinc-finger RNA-binding proteins. These proteins recognize AU-rich elements (AREs) in target mRNAs and promote deadenylation, decay, and translational repression. In this review, we use the term post-transcriptional immune checkpoint in a restricted conceptual sense: ZFP36 family proteins are intracellular, RNA-level negative regulators that tune the magnitude, duration, and resolution of immune effector programs, rather than classical receptor-ligand immune checkpoints such as programmed cell death protein 1 (PD-1)/ programmed death-ligand 1 (PD-L1) or cytotoxic T-lymphocyte-associated protein 4 (CTLA-4). We summarize structural features, ARE-recognition mechanisms, mRNA decay pathways, translational repression mechanisms, and post-translational regulation of the ZFP36 family, while explicitly distinguishing mechanisms established for ZFP36 from those inferred for ZFP36L1 and ZFP36L2. We then review cell-type-specific roles in innate and adaptive immunity, including myeloid inflammatory responses, barrier tissue inflammation, innate lymphoid cell function, T cell activation and effector differentiation, regulatory T cell stability, B cell development, and antiviral immunity. In cancer, ZFP36 family members show context-dependent functions that should be separated into tumor-cell-intrinsic effects and immune-microenvironment-dependent effects. They suppress tumor progression by destabilizing pro-inflammatory, angiogenic, metabolic, and epithelial–mesenchymal transition (EMT)-associated transcripts, yet may also restrict antitumor immune responses or promote immune evasion in selected tumor contexts. Finally, we discuss autoimmune and inflammatory diseases, allergic disorders, transplant immunity, neuroimmune relevance, and therapeutic strategies, emphasizing the current evidentiary limits, preclinical status, and safety concerns of ZFP36 family modulation.

## 1. Introduction

The immune response is a critical process by which the host recognizes pathogens, clears damaged cells, and restores tissue homeostasis. However, the intensity and duration of this response are determined not only by transcriptional activation but also by post-transcriptional mechanisms, including mRNA stability and translational efficiency [1,2,3,4]. Many immunity-associated mRNAs encoding cytokines such as tumor necrosis factor alpha (TNF-α), interleukin-6 (IL-6), interleukin-1 beta, and interferon gamma (IFN-γ) contain AU-rich elements (AREs) in their 3′ untranslated regions (3′ UTRs). These AREs serve as cis-acting elements recognized by specific RNA-binding proteins (RBPs) that promote deadenylation and mRNA decay, thereby limiting the expression of immune mediators [2,5,6]. 

The zinc finger protein 36 (ZFP36) family comprises three major members in mammals: ZFP36, ZFP36 CCCH-type like 1 (ZFP36L1), and ZFP36 CCCH-type like 2 (ZFP36L2). All three proteins contain a conserved tandem CCCH-type zinc finger domain that recognizes AREs within the 3′ UTRs of target mRNAs and recruits effector complexes such as the carbon catabolite repression 4–negative on TATA-less (CCR4–NOT) deadenylase complex, to promote mRNA deadenylation and translational repression [7,8]. Given that numerous transcripts encoding inflammatory cytokines, chemokines, and immunoregulatory molecules contain AREs, ZFP36 family proteins function as key post-transcriptional repressors of immune mediator expression [2,7,9], contributing to the resolution of inflammation and the maintenance of immune homeostasis [7,9].

In this review, we define the term “post-transcriptional immune checkpoint” as a regulatory framework describing intracellular mechanisms that restrain immune activation by controlling the stability, translation, and turnover of immune effector transcripts. Within this framework, ZFP36 family proteins differ from classical transcriptional regulators because they act after transcription has occurred [7,9,10]; they differ from canonical immune checkpoints such as programmed cell death protein 1 (PD-1)/programmed death-ligand 1 (PD-L1) and cytotoxic T-lymphocyte-associated protein 4 (CTLA-4) because they do not operate through cell-surface receptor-ligand inhibitory signaling [11]. They differ from many housekeeping RNA-binding proteins because their targets are enriched for inducible inflammatory, cytokine, chemokine, and immune-regulatory transcripts [4,7,10,11].

Their checkpoint-like behavior is characterized by several key features: they restrain immune effector programs at the post-transcriptional level [7,9]; their expression, phosphorylation, localization, and stability are dynamically regulated by inflammatory and activation-associated signals [12,13,14,15]; and they shape the magnitude, duration, and resolution of immune responses rather than functioning as binary switches [7,9]. Importantly, ZFP36 family proteins act as inducible restraints within immune regulatory networks, and their effects vary according to family member, target transcript, cell type, and tissue microenvironment [9,16,17]. This framework distinguishes ZFP36 family proteins from classical transcriptional regulators, canonical receptor–ligand immune checkpoints, and many RNA-binding proteins with broader roles in basal RNA metabolism or general RNA processing [4,18].

Although ZFP36, ZFP36L1, and ZFP36L2 share highly conserved RNA-binding domains and overlapping mechanisms of ARE-mediated mRNA decay, accumulating evidence indicates substantial divergence in cellular expression patterns, target selection, and disease-associated functions [9,16,17,19,20]. Throughout this review, we therefore emphasize not only their common post-transcriptional regulatory mechanisms but also the emerging themes of functional redundancy, cell-type specialization, and context-dependent biological activity.

This review focuses on the structural features, ARE recognition mechanisms, functions in mRNA decay and translational repression, and upstream regulatory mechanisms of ZFP36, ZFP36L1, and ZFP36L2. It further discusses their roles in innate immunity, adaptive immunity, tumor immunity, autoimmunity, allergic disease, transplant immunity, and neuroimmune regulation. Throughout the manuscript, we distinguish direct target-level evidence from pathway-level or correlative evidence and emphasize functional redundancy, cell-type specialization, and context-dependent biological activity.

## 2. Structural Organization and Molecular Mechanisms of ZFP36 Family Proteins

### 2.1. Domain Architecture and RNA-Recognition Features

In humans and most mammals, the ZFP36 family primarily comprises three widely expressed paralogs: ZFP36 (tristetraprolin, TTP/TIS11), ZFP36L1 (TIS11B/BRF1), and ZFP36L2 (TIS11D/BRF2) [9,21]. A fourth member, ZFP36L3, is restricted to rodent extraembryonic tissues (e.g., placenta and yolk sac) and lacks a clear human ortholog; consequently, it is not discussed in detail here [22,23,24].

ZFP36, ZFP36L1, and ZFP36L2 all contain a highly conserved tandem CCCH-type zinc finger (TZF) domain, composed of two CX_8_CX_5_CX_3_H motifs separated by an 18-amino-acid linker [25,26]. These TZF domains specifically recognize short AU-rich sequences within single-stranded RNA, preferentially binding class II AREs that contain the UUAUUUAUU nonamer; repeated or overlapping ARE motifs typically exhibit higher binding affinity [27,28]. The high conservation of the TZF domain underlies shared functions in ARE recognition and mRNA destabilization. In contrast, variations in non-conserved regulatory regions, cell-type contexts, upstream signaling pathways, and target mRNA accessibility further confer member-specific functions in distinct physiological and immune settings [9,29,30].

ZFP36 (encoded by mouse *Zfp36* and human *ZFP36*) is the most extensively studied family member. Its cDNA was first cloned and characterized by Varnum et al. in 1991 [31] from NIH 3T3 fibroblasts stimulated with serum or growth factors. The protein is named tristetraprolin due to its multiple PPPP tetraproline motifs [31]. ZFP36 has an approximate molecular mass of 34 kDa, and the human *ZFP36* gene is located on chromosome 19q13.2. In inflammatory contexts, *ZFP36* mRNA expression is rapidly and transiently induced by stimuli such as lipopolysaccharide (LPS) and TNF-α, consistent with its classification as an immediate-early gene (IEG) [31,32]. In macrophages, dendritic cells, and activated T cells, ZFP36 limits the persistent expression of pro-inflammatory mediators [5,7,33]. Mice lacking *Zfp36* exhibit a systemic inflammatory syndrome characterized by markedly elevated plasma TNF-α, accompanied by arthritis, dermatitis, and autoimmune-like manifestations, suggesting that ZFP36 is a key negative regulator of *Tnf* mRNA stability [32,34].

ZFP36L1 is encoded by the human *ZFP36L1* gene on chromosome 14q24.1 and has an approximate molecular mass of 36.3 kDa. Compared with ZFP36, ZFP36L1 retains a conserved tandem CCCH-type zinc finger domain, whereas its N- and C-terminal non-zinc-finger regions differ in length and sequence. These differences suggest that ZFP36L1 mediates mRNA decay and post-transcriptional regulation through distinct protein-interaction networks [21,35]. ZFP36L1 plays an important role in the post-transcriptional regulation of cell cycle-related mRNAs and, together with ZFP36L2, contributes to the maintenance of lymphocyte quiescence. During development, *Zfp36l1* knockout induces defects in chorioallantoic fusion and results in embryonic lethality. Additionally, ZFP36L1 has also been reported to regulate vascular endothelial growth factor (VEGF) expression and angiogenesis [17,19,36,37]. 

ZFP36L2 is encoded by the human *ZFP36L2* gene on chromosome 2p21 and has an approximate molecular mass of 51 kDa. Compared with ZFP36 and ZFP36L1, ZFP36L2 exhibits more pronounced functional specificity across developmental stages and cell types [38,39,40,41,42]. ZFP36L2 is highly expressed in oocytes and early embryos and is involved in oocyte maturation, the maintenance of female fertility, and early embryonic development [39,41]. Recent studies indicate that ZFP36L2 attenuates IFN-γ expression and modulates T cell effector functions during certain immune responses [16,33]. The basic characteristics of the three human ZFP36 family RNA-binding proteins are summarized in Table 1.

**Figure 1:**
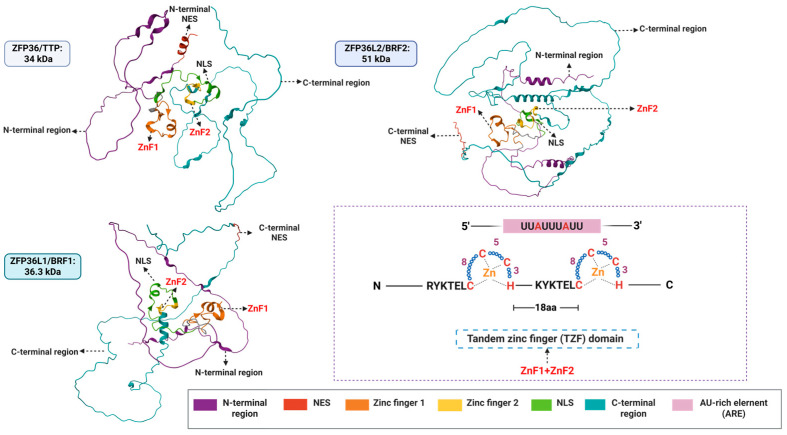
Conserved domain architecture and AU-rich element (ARE) recognition of ZFP36 family proteins. ZFP36, ZFP36L1, and ZFP36L2 share a highly conserved tandem CCCH-type zinc finger (TZF) domain that mediates sequence-specific recognition of ARE-containing target mRNAs and forms the structural basis for post-transcriptional regulation. The nuclear export signal (NES) and nuclear localization signal (NLS) regulate nucleocytoplasmic trafficking, whereas the N-terminal and C-terminal regions provide regulatory interfaces for protein interactions and post-translational modifications, thereby influencing protein stability and activity. The inset illustrates the tandem arrangement of zinc finger 1 (ZnF1) and zinc finger 2 (ZnF2) within the TZF domain and their recognition of the consensus ARE motif in target mRNAs. These domain features link RNA recognition to downstream mRNA decay, translational repression, and context-dependent functional specialization. Colors correspond to the domain key shown below the figure. In the inset, the red ZnF1 and ZnF2 labels identify the two zinc fingers. C and H indicate the conserved zinc-coordinating residues, Zn denotes a zinc ion, and the numbers 8, 5, and 3 indicate the numbers of intervening amino acid residues in the C-X8-C-X5-C-X3-H motif. N and C denote the protein termini, 5′ and 3′ indicate the RNA orientation, and aa denotes amino acids. Created in BioRender. Luo, L. (2026) https://BioRender.com/fhbr2tw (accessed on 7 July 2026).

The conserved domain organization and ARE-recognition features of ZFP36 family members are summarized in Figure 1, highlighting their shared TZF-mediated RNA-binding module as well as member-specific structural differences that may contribute to functional specialization.

### 2.2. ARE-Mediated mRNA Decay and Translational Control

Building on the structural overview shown in Figure 1, this section summarizes how TZF-mediated ARE recognition is coupled to mRNA deadenylation, decay, translational repression, and signal-dependent regulation, as ZFP36 serves as the mechanistic prototype for the family. The mechanisms described below are presented primarily as experimentally established for ZFP36. ZFP36L1 and ZFP36L2 share conserved RNA-binding and NOT1-interacting features, but their mechanisms, targets, and regulatory partners should not be assumed to be identical unless direct evidence is available.

#### 2.2.1. Deadenylation, Decapping, Exonucleolytic Decay, and P-Body Association

Upon AREs binding, ZFP36 recruits the CCR4-NOT deadenylase complex via its C-terminal CNOT1-binding motif. This interaction leads to poly(A) tail shortening by the catalytic subunits CNOT6/6L and CNOT7/8, thereby initiating mRNA decay [30,43]. Recent evidence indicates that multiple low-complexity regions in ZFP36 mediate multivalent interactions with CCR4-NOT and poly(A)-binding protein cytoplasmic 1 (PABPC1), which enhances deadenylation efficiency [8].

Following deadenylation, mRNAs that have lost poly(A)-tail-mediated protection undergo DCP1/DCP2-mediated decapping of the 5′ 7-methylguanosine (m^7^G) cap. This process exposes the 5′ monophosphate end to degradation by exoribonuclease 1 (XRN1) in the 5′-3′ direction. Meanwhile, a subset of deadenylated mRNAs is recognized by the cytoplasmic RNA exosome and its auxiliary superkiller (SKI) complex, leading to degradation in the 3′-5′ direction [35,43]. Notably, the N-terminal intrinsically disordered region of ZFP36 directly interacts with DCP2, facilitating decapping [35].

Processing bodies (P-bodies) contain abundant mRNA silencing and decay factors, including DCP1/DCP2, XRN1, DEAD-box helicase 6 (DDX6), and eIF4E transporter (4E-T). Certain ZFP36 target mRNAs and ZFP36-containing ribonucleoprotein complexes colocalize with P-body components [35,44]. And ZFP36-mediated mRNA decay is functionally associated with P-bodies but is not strictly dependent on microscopically visible P-body structures [44].

#### 2.2.2. Translational Repression and Stress-Granule Dynamics

In addition to promoting mRNA degradation, ZFP36 diminishes the efficiency of target mRNA translation. For mRNAs encoding short-lived inflammatory mediators such as TNF-α, translational repression and mRNA destabilization act together to restrict their expression [7,8,29]. p38 mitogen-activated protein kinase (MAPK)–MAPK-activated protein kinase 2 (MK2) signaling regulates the competitive binding of ZFP36 and human antigen R (HuR) to AREs of *TNF* mRNA by phosphorylating ZFP36. Upon binding to 14-3-3 proteins, phosphorylated ZFP36 exhibits reduced repressive activity, thereby allowing HuR to maintain translational activity [12,13]. Conversely, dephosphorylated ZFP36 binds more readily to AREs, promoting the transition of target mRNAs from a translationally active state to a silenced, decay-prone state [45]. ZFP36 recruits the atypical cap-binding protein eukaryotic translation initiation factor 4E family member 2 (eIF4E2)/ eIF4E-homologous protein (4EHP) to ARE-containing mRNAs to inhibit their translation. Moreover, the tetraproline motifs of ZFP36 promote translational repression and subsequent decay of ARE-containing mRNAs by forming the 4EHP-GRB10-interacting GYF protein 2 (GYF2) complex [46,47]. 

Stress granules (SGs) are cytoplasmic condensates that concentrate translationally stalled mRNAs and translation initiation factors, and they may participate in regulating translation under stress [48]. ZFP36 is recruited to SGs induced by energy deprivation. Moreover, p38-MK2-mediated phosphorylation of ZFP36 promotes its binding to 14-3-3 proteins, thereby modulating its association with SGs [12].

#### 2.2.3. Post-Translational Regulation of ZFP36 Activity and Stability

ZFP36 is regulated by transcription as well as by post-translational modifications that modulate its protein activity and stability. The phosphorylation by the p38 MAPK-MK2 axis is the most extensively studied. Inflammatory stimuli, such as LPS, TNF-α, and IL-1β, activate p38 MAPK and the downstream kinase MK2, which subsequently phosphorylate critical serine residues in ZFP36 [7,14,29]. The primary phosphorylation sites in murine ZFP36 are Ser52 and Ser178, whereas the corresponding sites in human ZFP36 are Ser60 and Ser186 [13,14]. Additionally, kinases such as extracellular signal-regulated kinase (ERK) and protein kinase R (PKR) have been reported to phosphorylate ZFP36 in response to specific stimuli or in particular cells. This suggests that ZFP36 serves as a regulatory node integrating inflammation-, stress-, and proliferation-related signals [14].

Beyond phosphorylation, ZFP36 protein abundance is regulated by the ubiquitin-proteasome system. A 2023 study indicates that ZFP36 undergoes lysine ubiquitination and subsequent proteasomal degradation [15]. These findings indicate that ZFP36 activity is determined not only by its ARE-binding capacity but also by the ZFP36 stability, which is jointly regulated by phosphorylation, 14-3-3 binding, and ubiquitination [7,15].

Overall, ZFP36-mediated processes, including ARE recognition, CCR4-NOT recruitment, deadenylation, decapping, translational repression, and signal-dependent regulation, provide a mechanistic framework for understanding the family [7,8,14,30]. ZFP36L1 and ZFP36L2 also possess conserved TZF domains and can promote ARE-containing mRNA destabilization, but their expression patterns, induction kinetics, interaction partners, and target repertoires are more cell-type and developmental-stage-dependent [9,19,38]. For example, ZFP36L1 and ZFP36L2 are central to lymphocyte quiescence, T cell fitness, Treg stability, and developmental programs, whereas ZFP36 is often a rapidly induced inflammatory feedback regulator [16,17,19]. Therefore, subsequent sections explicitly indicate whether evidence is direct target-level evidence, combined genetic evidence, pathway-level evidence, or inference based on family conservation. 

## 3. ZFP36 Family Proteins in Innate Immunity

Extending these mechanistic insights to innate immune biology, myeloid cells, ILCs, and epithelial barriers deploy ZFP36 family proteins as RNA-level regulators of inflammatory restraint. These compartments form the first line of defense against invading pathogens and tissue injury, but excessive or persistent activation can cause tissue damage and systemic inflammation [2,49,50,51,52]. In this setting, ZFP36 family proteins act as post-transcriptional checkpoint-like factors because they terminate or attenuate inflammatory gene expression after transcriptional induction by shortening the half-life of selected cytokine and chemokine transcripts [1,7,11,20,29,32]. This section therefore discusses not only individual studies but also shared regulatory principles and cell-type-specific differences across myeloid cells, innate lymphoid cells (ILCs), and barrier tissues.

### 3.1. Myeloid Inflammatory Responses

Myeloid cells are central effectors of innate immunity and are strongly shaped by ZFP36 family post-transcriptional regulation during inflammation [20]. Upon sensing pathogen- or tissue damage-derived signals, macrophages, dendritic cells, and neutrophils rapidly induce TNF-α, IL-6, IL-1β, and various chemokines to promote pathogen clearance and inflammatory-cell recruitment [51,53,54]. In these cells, ZFP36 family proteins function as key negative-feedback regulators that shorten the half-life of selected inflammatory transcripts, thereby preventing excessive amplification of cytokine and chemokine programs [20,55].

*Zfp36*-deficient mice develop a systemic inflammatory phenotype characterized by cachexia, arthritis, dermatitis, and myeloid hyperplasia, associated with markedly elevated TNF-α levels [34]. ZFP36 was subsequently shown to provide feedback inhibition of macrophage TNF-α production by destabilizing *Tnf* mRNA [32]. This regulatory mechanism is not restricted to TNF-α alone. ZFP36-mediated mRNA decay can target multiple inflammation-induced transcripts and thereby regulate both the magnitude and timing of acute inflammatory responses [56]. Following LPS stimulation, the three ZFP36 family members show distinct expression and regulation. ZFP36 is rapidly induced, whereas *Zfp36l1* and *Zfp36l2* mRNAs are markedly downregulated in RAW264.7 macrophages; ZFP36L1 and ZFP36L2 proteins nevertheless localize mainly in the cytoplasm and undergo rapid phosphorylation [57]. These observations suggest partial overlap but not simple redundancy. ZFP36 functions as a dominant inducible feedback regulator during acute stimulation, whereas ZFP36L1 and ZFP36L2 may contribute to basal transcript homeostasis, buffering, or compensation depending on cellular state [9,57].

Studies of dendritic cells and neutrophils further indicate that ZFP36-mediated regulation differs across myeloid subsets. In bone marrow-derived dendritic cells (BMDCs), ZFP36 deficiency markedly enhances LPS-induced IL-23 production, indicating that ZFP36 limits IL-23-associated inflammatory signaling in antigen-presenting cells [58]. In neutrophils, ZFP36 promotes post-infection apoptosis by reducing *Mcl1* mRNA stability and MCL1 protein expression; conversely, myeloid cell-specific *Zfp36* deletion prolongs neutrophil survival, increases their accumulation at infection sites, and enhances antibacterial activity [59]. These findings suggest that ZFP36 regulates myeloid inflammation through distinct effector programs, predominantly controlling cytokine production in dendritic cells and cell survival in neutrophils [58,59].

Combined deletion studies have further revealed functional synergy among ZFP36 family members. Deleting *Zfp36*, *Zfp36l1*, or *Zfp36l2* individually produces relatively limited spontaneous inflammatory phenotypes, whereas simultaneous deletion of all three genes in myeloid cells causes severe systemic inflammation, arthritis-like lesions, and premature death, accompanied by increased stability of transcripts encoding proinflammatory cytokines and chemokines [20].

Taken together, available evidence supports a cooperative but non-identical model of ZFP36 family function. A common regulatory principle is ARE-dependent shortening of inflammatory-transcript half-life after transcriptional induction, which prevents cytokine and chemokine programs from becoming self-amplifying. Functionally, however, ZFP36 is best supported as a rapidly inducible feedback regulator of acute macrophage and dendritic-cell cytokine production, whereas ZFP36L1 and ZFP36L2 appear to provide basal buffering, compensation, or phase-specific control that becomes most evident in combined-deletion models [9,20,32,56,57]. The key unresolved questions are which direct targets are uniquely controlled by ZFP36L1 or ZFP36L2 in each myeloid subset, how phosphorylation changes their target selection, and whether these proteins differentially regulate cytokine production, chemokine recruitment, and survival programs. Biologically, this layered regulation allows myeloid cells to mount rapid antimicrobial and tissue-repair responses while limiting systemic inflammatory amplification [20,58,59]. 

### 3.2. Innate Lymphoid Cell Responses

Similar to myeloid cells, ILCs lack antigen-specific receptors but produce effector cytokines in response to tissue microenvironmental stimuli. Among ILC subsets, group 2 innate lymphoid cells (ILC2s) are major mediators of type 2 immune responses and constitute a primary source of interleukin-5 (IL-5) and interleukin-13 (IL-13) in mucosal tissues, contributing to anti-helminth immunity, allergic inflammation, and the maintenance of intestinal eosinophil homeostasis [60,61]. ZFP36 is highly expressed in resting ILC2s but is rapidly downregulated following interleukin-33 (IL-33) stimulation. ZFP36 overexpression suppresses IL-5 and IL-13 production, whereas *Zfp36* deficiency augments ILC2-derived IL-5 and IL-13 [62]. Mechanistically, ZFP36 directly targets AREs within the 3′ UTR of *Il5* mRNA and reduces its stability, whereas IL-13 regulation appears indirect [62]. *Zfp36* deficiency also promotes local expansion of interleukin-22 (IL-22)^+^ group 3 innate lymphoid cells (ILC3s) in the intestinal lamina propria, and *Il22* deficiency mitigates dextran sulfate sodium (DSS)-colitis susceptibility in *Zfp36*-deficient mice [63]. At present, ILC evidence indicates that ZFP36 family regulation extends beyond classical myeloid inflammation to cytokine programs driven by tissue cues. The common principle is again transcript-level restraint of inducible effector cytokines, but the functional outcome differs by ILC subset: in ILC2s, ZFP36 directly limits *Il5* mRNA stability and indirectly constrains IL-13 output, whereas in intestinal ILC3s it modulates an IL-22-associated inflammatory axis whose direct RNA targets remain undefined [62,63]. Direct target validation for ZFP36L1 and ZFP36L2 in ILC subsets is still lacking, making ILC1-, ILC2-, and ILC3-specific deletion and CLIP-based target mapping important next steps.

### 3.3. Barrier-Tissue Innate Immunity

Epithelial cells in the skin, airways, and intestine are crucial to innate immune defense. Beyond forming physical barriers, they actively shape the local immune microenvironment by secreting cytokines, chemokines, and antimicrobial molecules [64,65,66]. In skin, ZFP36 dampens keratinocyte-derived inflammatory amplification by reducing the stability of inflammation-associated mRNAs, including *Tnf*, *Cxcl1*, and *Cxcl2*; keratinocyte-specific *Zfp36* deletion causes psoriasis-like skin inflammation, arthritis-like pathology, and systemic inflammation [67]. In chemical skin carcinogenesis, ZFP36 directly targets *Areg* mRNA, thereby limiting aberrant activation of epidermal growth factor receptor (EGFR) signaling and suppressing tumor-associated inflammation and epidermal tumor formation [68]. In psoriatic fibroblasts, *ZFP36* promoter/coding-region methylation is associated with reduced *ZFP36* expression and enhanced NOD-, LRR- and pyrin domain-containing protein 3 (NLRP3) inflammasome activation [69]. And in the airway epithelium, ZFP36 promotes *C-X-C motif chemokine ligand 8* (*CXCL8*) mRNA (encoding interleukin-8, IL-8) deadenylation and degradation in cystic fibrosis lung epithelial cells [70]. By contrast, the reported downregulation of ZFP36L1 and ZFP36L2 in severe asthma airway epithelium remains largely correlative; specific target mRNAs and causal mechanisms require validation [71]. In intestinal epithelial cells, *Zfp36* deletion reduces susceptibility to DSS-induced acute colitis, illustrating that ZFP36 activity can have disease- and tissue-stage-specific consequences rather than uniformly anti-inflammatory effects [72].

Viewed across barrier tissues, ZFP36 family activity does not function as a uniform anti-inflammatory switch. Instead, it tunes local epithelial-immune circuits by controlling the persistence of selected transcripts and the timing of tissue damage, repair, and inflammatory recruitment. In skin and airway epithelium, ZFP36 predominantly restrains cytokine, chemokine, and growth-factor programs such as *Tnf*/*Cxcl1*/*Cxcl2*, *Areg*, and *CXCL8*, thereby limiting inflammatory amplification and tumor-promoting signaling [67,68,70]. In the intestine, however, epithelial *Zfp36* deletion can reduce DSS-colitis susceptibility, illustrating that transcript decay may either protect or exacerbate pathology depending on the dominant cellular source and disease phase [72]. The major gaps are the direct epithelial targets of ZFP36L1/ZFP36L2 and the conditions under which ZFP36-mediated decay favors barrier repair versus inflammatory resolution [71,72]. Overall, innate immune data support the concept of ZFP36 proteins as tunable post-transcriptional checkpoints whose biological significance depends on immune-cell identity, barrier context, and inflammatory timing.

To clarify the distinction between experimentally validated mechanisms and inferred or pathway-level associations, we summarized disease contexts, involved cell types, representative targets or pathways, mechanistic evidence, and functional outcomes of ZFP36 family members in Table 2.

## 4. ZFP36 Family Proteins in Adaptive Immunity

Compared with innate immune responses, adaptive immunity depends more on antigen-specific recognition, clonal expansion, and effector differentiation. Dysregulation of these responses may disrupt the balance among pathogen clearance, tumor immune surveillance, and immunopathological damage [92,93]. Within adaptive immunity, ZFP36 family proteins participate in several regulatory steps, including the transition from quiescence to activation, effector differentiation, Treg stability, B cell development, and humoral responses. Specifically, ZFP36 decreases the expression of effector molecules such as TNF-α and IFN-γ following T cell activation [73]; whereas ZFP36L1 and ZFP36L2 are implicated in T cell quiescence, antigen-specific clonal expansion, Treg cell stability, B cell development, and humoral immune responses [16,17,78,79,80]. Consequently, ZFP36 family proteins should not be viewed merely as suppressors of adaptive immunity; rather, they contribute to the post-transcriptional control of the magnitude and duration of lymphocyte responses [16,17,73,78,79,80].

### 4.1. T Cell Activation, Effector Differentiation, and Regulatory T Cell Stability

T cell activation involves a rapid transition from quiescence to proliferation and effector differentiation. This process requires the swift induction of activation markers, cytokines, and cell cycle-related genes, while preventing excessive amplification of effector responses [93,94,95]. ZFP36 restrains effector cytokine expression following T cell activation. Upon peptide stimulation, *Zfp36*-deficient T cells exhibited elevated production of TNF-α and IFN-γ, an increased proportion of TNF-α/IFN-γ double-positive effector cells, and enhanced T cell expansion [73]. Following T cell receptor (TCR) stimulation, ZFP36 and ZFP36L1 proteins rapidly accumulate, peaking during the early phase of CD4^+^ and CD8^+^ T cell activation. This suggests that they function during the early stage of T cell activation to hinder the expression of activation-associated mRNAs [73,74].

ZFP36 targets activation- and effector-associated mRNAs, including *Cd69*, *Il2*, *Ifng*, and *Tnf*, thereby decreasing the corresponding protein expression. Consistently, *Zfp36*-deficient CD4^+^ T cells display enhanced CD69, IFN-γ, and TNF-α expression shortly after activation, along with increased proliferation and apoptosis [73]. Dual deletion of *Zfp36* and *Zfp36l1* further accelerates the transition of CD8^+^ T cells toward an effector state and enhances cytotoxic function [74]. These findings indicate that ZFP36 family proteins do not merely block T cell activation; rather, they restrict the amplitude and duration of activation-marker, cytokine, and differentiation-related transcript expression during early activation [73,74].

The ZFP36 family members exhibit both functional overlap and temporal specialization in maintaining T cell quiescence and regulating effector differentiation. Genetic models indicate that loss of individual family members usually produces modest phenotypes, whereas combined deletion causes more pronounced dysregulation of T cell activation, supporting partial redundancy [16]. However, individual family members also operate at distinct stages and signaling contexts during T cell responses. ZFP36 and ZFP36L1 are rapidly induced following TCR stimulation and have been implicated in constraining early activation-associated mRNA expression programs by limiting the expression of activation- and cytokine-associated transcripts [73,74]. ZFP36L1 also regulates TCR affinity-dependent CD8^+^ T cell responses by linking TCR affinity to interleukin-2 (IL-2) responsiveness and preferential expansion of high-affinity cells [75]. In contrast, ZFP36L2 exerts a more time-dependent regulatory effect. T cell-specific deletion of *Zfp36l2* has little effect during the first 2–6 h after activation but increases IFN-γ production after 20–48 h [33]. Furthermore, dual deletion of *Zfp36l1* and *Zfp36l2* increases cytokine production but compromises viability, proliferation, and cell-cycle control, indicating that these proteins balance effector output with T cell fitness [76]. 

Regulatory T cells (Tregs) are essential for maintaining peripheral immune tolerance and preventing autoimmune responses. Impaired Treg function or aberrant expression of inflammatory cytokines, such as IFN-γ, by Tregs can compromise their immunosuppressive capacity and promote inflammation [96,97,98]. The Treg-specific genetic model, in which *Zfp36l1* and *Zfp36l2* in Tregs are simultaneously deleted, leads to spontaneous inflammation and is accompanied by elevated serum inflammatory cytokines and immunoglobulins, suggesting impaired Treg-mediated immunosuppression [17]. Furthermore, the inflammatory phenotype caused by ZFP36L1/ZFP36L2 deficiency in Tregs is not primarily due to direct impairment of forkhead box P3 (FOXP3) expression but is associated with aberrant enhancement of IFN-γ-related pathways. In the double-deficient context, deletion of a single *Ifng* allele markedly alleviated inflammatory pathology [17,99]. Double-deficient Tregs exhibit reduced CTLA-4 recycling, decreased sensitivity to survival signals, such as IL-2 and interleukin-7 (IL-7), and enhanced responsiveness to IFN-γ signaling. These changes mitigate their ability to suppress antigen-presenting cells, such as conventional type 2 dendritic cells (cDC2s), thereby further promoting the expansion of effector T cell and T follicular helper (Tfh) cell responses [17,100,101,102]. In addition to acting with ZFP36L1 to maintain Tregs homeostasis, ZFP36L2 regulates inducible Treg (iTreg) differentiation by targeting *Ikaros family zinc finger 2* (*Ikzf2*) mRNA. Conversely, ZFP36L2 overexpression reduces Helios levels and impairs the suppressive function of iTregs [77].

Integrating these T cell and Treg findings reveals both shared principles and divergent functions. The shared principle is post-transcriptional buffering of activation-induced mRNAs, which prevents short-lived effector transcripts from remaining expressed after the initial stimulus. The functional division is temporal and cell-context dependent: ZFP36 and ZFP36L1 act prominently during early conventional T cell activation; ZFP36L1 links TCR affinity to IL-2 responsiveness and high-affinity CD8^+^ T cell expansion; ZFP36L2 is more evident during later effector phases, especially in limiting IFN-γ production; and ZFP36L1/ZFP36L2 together maintain Treg suppressive stability through IFN-γ-related pathways, CTLA-4 recycling, cytokine responsiveness, and Helios regulation [17,33,73,75,76,77]. Unresolved issues include the direct member-specific target sets in distinct T cell states, how target selection changes during acute versus chronic antigen stimulation, and whether selective modulation can enhance antiviral or antitumor responses without impairing Treg-mediated tolerance.

### 4.2. B Cell Development and Humoral Immunity

B cells develop from bone marrow precursors into mature peripheral subsets and, upon antigen stimulation, undergo germinal center responses and differentiate into antibody-secreting cells, the plasma cells, and the memory B cells [103,104,105]. Among ZFP36 family members, ZFP36L1 and ZFP36L2 contribute to early B cell development, marginal zone B cell (MZ B cell) maintenance, germinal center responses, and plasma cells homing by regulating mRNAs involved in cell-cycle control, migration, localization, and subset maintenance [19,78,79,80].

ZFP36L1 and ZFP36L2 exhibit substantial functional overlap during highly proliferative stages of B cell differentiation. Combined deletion of *Zfp36l1* and *Zfp36l2* causes abnormal proliferation of precursor B cells and disrupts the cell-cycle control required for immunoglobulin gene rearrangement [19]. Both proteins are also critical for germinal-center B cell cycle progression and maintenance of germinal-center responses; their depletion increased DNA replication stress, accumulation of DNA damage, and G2-M arrest [79]. These findings suggest that the redundant functions of ZFP36L1 and ZFP36L2 are particularly important in maintaining genomic stability during periods of rapid B cell proliferation. By contrast, ZFP36L1 appears to have more specialized roles in mature compartments: B cell-specific *Zfp36l1* deletion reduces marginal zone B cells, and ZFP36L1 deficiency impairs plasma-cell homing to bone marrow, causing abnormal accumulation of plasma cells in spleen and liver [78,79]. These observations indicate that, beyond its cooperative functions with ZFP36L2, ZFP36L1 also contributes to the spatial organization and maintenance of mature humoral immune compartments. 

Overall, B-cell data illustrate a stage-dependent shift from redundancy to specialization. ZFP36L1 and ZFP36L2 cooperate during precursor and germinal-center stages, where the common regulatory principle is restraint of cell-cycle and replication-stress-associated transcript programs to preserve quiescence, orderly immunoglobulin rearrangement, and genomic stability [19,80]. In mature humoral compartments, ZFP36L1 appears more specialized, supporting marginal-zone B cell maintenance and plasma-cell homing to bone marrow [19,78,79,80]. The major unresolved questions are which transcripts are direct versus indirect targets at each developmental stage, how ZFP36L1-specific functions are separated from ZFP36L2 compensation, and whether these RNA-level checkpoints influence antibody quality, memory formation, or autoantibody generation.

### 4.3. Antiviral T Cell Responses and Immunopathology

During infection, T cells rapidly reestablish a balance between pathogen clearance and the prevention of excessive inflammatory damage [106,107]. ZFP36 family proteins fine-tune the magnitude and duration of anti-infective responses [73,74]. In an lymphocytic choriomeningitis virus (LCMV) Armstrong infection model, *Zfp36*-deficient mice display accelerated viral clearance accompanied by enhanced virus-specific CD4^+^ and CD8^+^ T cell responses [73]. 

Similarly, in an influenza A virus infection model, adoptive transfer of a small number of CD8^+^ T cells lacking both *Zfp36* and *Zfp36l1* enhances host protection against viral infection, without inducing obvious immunopathological damage [74]. Following chronic antigen stimulation or in tumor-infiltrating T cells, *Zfp36l2* deficiency enhances IFN-γ production. In contrast, dual *Zfp36l1*/*Zfp36l2* deficiency increases T cell cytokine production but is associated with reduced viability, impaired proliferation, and cell-cycle dysregulation [33,76].

Beyond modulating host T cell responses, ZFP36L1 directly affects viral replication. It targets the hemagglutinin (HA), matrix (M), and nonstructural (NS) RNA segments of the influenza A virus, thereby suppressing viral protein translation. Moreover, ZFP36L1 restricts flavivirus infection by promoting XRN1-mediated 5′-3′ RNA decay and RNA exosome-mediated 3′-5′ RNA decay [81,82].

Taken together, antiviral studies emphasize that ZFP36 family proteins act at two biologically distinct levels. In immune cells, ZFP36 and ZFP36L1 limit the magnitude and timing of T cell effector differentiation, whereas ZFP36L2 constrains late or sustained IFN-γ production [33,73,74]. In infected target cells, ZFP36L1 can directly restrict viral RNA translation or stability, as shown for influenza and flavivirus models [81,82]. This dual action raises an important unresolved question: whether therapeutic modulation should be directed toward immune cells, infected cells, or both. The biological significance is therefore context dependent: decreasing ZFP36 family restraint may accelerate T cell-mediated viral clearance, whereas enhancing viral RNA decay may suppress replication; either strategy must be balanced against the risk of immunopathology, impaired T cell fitness, or loss of tissue tolerance.

## 5. Context-Dependent Roles of ZFP36 Family Proteins in Cancer

ZFP36 family proteins also balance immune activation and immunopathology in cancer, where they exhibit context-dependent functions in tumorigenesis and tumor progression through shared inflammatory and immune pathways [108,109]. These roles should be separated into tumor-cell-intrinsic mechanisms and immune-microenvironment-dependent mechanisms. In many tumor cells, ZFP36 family members suppress tumor progression by destabilizing mRNAs encoding pro-inflammatory mediators, proliferation-related genes, angiogenic factors, metabolic enzymes, or epithelial–mesenchymal transition (EMT)-associated transcription factors [68,108,109,110,111]. In the tumor microenvironment, however, the same RNA-destabilizing logic may restrain cytokines and effector molecules required for antitumor immunity. Thus, ZFP36 family activity can either suppress tumor-promoting inflammation and malignant phenotypes or limit immune-mediated tumor control, depending on tumor type, cellular source, disease stage, and immune context [16,33,85,95].

### 5.1. Tumor-Cell-Intrinsic Tumor-Suppressive Mechanisms

Tumor-cell-intrinsic antitumor effects are supported by studies showing that ZFP36 family members destabilize transcripts linked to inflammatory signaling, proliferation, angiogenesis, EMT, metabolism, and chemoresistance. In several solid tumors, downregulation or functional impairment of ZFP36 weakens repression of inflammatory mediators such as TNF and IL-6, thereby supporting persistent inflammatory signaling and tumor progression [112]. Restoration of ZFP36 expression can suppress nuclear factor kappa B (NF-κB)/p65 activity and inflammation-related NF-κB-IL-6- signal transducer and activator of transcription 3 (STAT3) signaling, reducing tumor-promoting inflammation [113,114,115,116].

At the cancer-cell level, ZFP36 destabilizes transcripts associated with the cell cycle, angiogenesis, and EMT. Loss of ZFP36 leads to the accumulation of proliferation-related transcripts, such as *CCND1* and *MYC*, as well as the angiogenic factor VEGF, thereby promoting proliferation, invasion, and angiogenic capacity, whereas restoration of ZFP36 suppresses these malignant phenotypes through downregulation of the same transcript network in non-small-cell lung cancer (NSCLC) models [112,117,118]. ZFP36 has also been reported to reverse chemoresistance in NSCLC by disrupting lipid-droplet accumulation through the regulation of *fatty acid synthase* (*FASN*) mRNA [83]. In liver cancer cells, ZFP36L1 binds the ARE-containing 3′ UTR of *ZEB2* mRNA and promotes its degradation, thereby suppressing EMT, migration, and invasion [84]. In thymocytes, ZFP36L1 and ZFP36L2 act redundantly to enforce the thymic β-selection checkpoint by limiting DNA damage response signaling and cell-cycle progression, supporting their role in maintaining lymphoid developmental homeostasis [119]. 

In addition to regulating proliferation and EMT, ZFP36 influences tumor progression by regulating tumor metabolism and chemoresistance. In NSCLC, ZFP36 binds to the ARE in the 3′ UTR of *FASN* mRNA, promoting its degradation and inhibiting lipid droplet (LD) accumulation. This process reverses chemotherapy resistance and suppresses tumor progression; furthermore, it enhances antitumor efficacy with chemotherapy combination [83]. In multiple myeloma, the long non-coding RNA (lncRNA) *PLUM* suppresses the transcription of tumor suppressor genes, such as *ZFP36*, by enhancing PRC2/EZH2-mediated H3K27me3 modification and activating the unfolded protein response (UPR) pathway, ultimately promoting chemoresistance. Targeting the *PLUM*-EZH2 interaction restores *ZFP36* expression and reverses this resistance [120].

Non-coding RNA networks can also enhance the tumor-suppressive effects of ZFP36 by restoring its expression. The circular RNA (circRNA)_000554 in breast cancer functions as a competing endogenous RNA (ceRNA), binding miR-182 and thereby alleviating the inhibitory effect on the 3′ UTR of *ZFP36* mRNA. This promotes E-cadherin expression, reduces the migratory and invasive capabilities of breast cancer cells, inhibits tumor growth in vivo, and induces autophagy and apoptosis [111].

Collectively, tumor-cell-intrinsic functions of the ZFP36 family are mainly reflected in the regulation of proliferation, angiogenesis, EMT, metabolism, chemoresistance, and malignant transformation. In many tumor models, these effects support a tumor-suppressive role for ZFP36 family members; however, this conclusion should not be generalized across all cancers because the direction of effect depends on family member, tumor lineage, immune context, and disease stage.

### 5.2. Immune-Microenvironment-Dependent Effects in Cancer

Building on tumor-cell-intrinsic functions, ZFP36 family proteins also regulate tumor immunity through immune-microenvironment-dependent mechanisms, influencing immune effector cells, regulatory T cells, macrophages, cytokine networks, and immune-evasion pathways [16,67,95]. These effects are highly context dependent. In T cells, ZFP36 family proteins restrain activation- and cytokine-associated transcripts, including those encoding IFN-γ, TNF, and IL-2, which may weaken CD8^+^ T-cell-mediated antitumor immunity in a specific context [16,33,73,95]. In Tregs, ZFP36L2-mediated regulation of *Ikzf2*/Helios may reduce suppressive function and thereby support antitumor immunity under selected conditions [77]. In tumor cells, ZFP36L1-driven regulation of the histone deacetylase 3 (HDAC3)–PD-L1 axis can facilitate immune escape, linking tumor-cell-intrinsic RNA regulation to immune-microenvironment-dependent effects [85]. 

Regulatory T cells represent one immune compartment in which ZFP36 family members may affect antitumor immunity. ZFP36L2 downregulates *Ikzf2* expression in Treg cells, thereby attenuating their immunosuppressive function and reducing their inhibition of effector T cells, which enhances the host antitumor immune response [77].

In addition to these tumor-cell-intrinsic mechanisms, ZFP36 family members may also promote tumor progression through immune-microenvironment-dependent regulation. In the context of immunotherapy, the anti-inflammatory and immunosuppressive functions of the ZFP36 family may attenuate antitumor immune responses. In effector T cells, ZFP36 family proteins hinder the expression of key effector molecules, such as IFN-γ, TNF, and IL-2. In T cells, the ZFP36 family influences differentiation and the cytotoxic function of CD8^+^ T cells, thereby reducing responsiveness to immune checkpoint inhibitor therapy [16,95]. This suggests that while the ZFP36 family mitigates tumor progression by suppressing inflammation, it may act as a restrictive factor for immune-dependent antitumor responses [121].

Macrophages provide another example of immune-microenvironment-dependent regulation. *Zfp36* deficiency does not directly affect the intrinsic function of CD8^+^ T cells; rather, it indirectly enhances the activation, proliferation, and IFN-γ secretion of cytotoxic T lymphocytes (CTLs) via a macrophage paracrine pathway. Specifically, *Zfp36* deficiency leads to excessive IL-27 release from macrophages, which activates CTLs, enhances tumor immune surveillance, and inhibits mammary tumor growth in mice [122].

Some immune-microenvironment-dependent effects originate from tumor-cell-intrinsic RNA regulation that alters immune-evasion pathways. In invasive gastric cancer cells, ZFP36L1 is a key RNA-binding protein upregulated by super-enhancers (SEs). Upon activation, ZFP36L1 directly binds to the ARE in the 3′ UTR of *HDAC3* mRNA, promoting degradation and decreasing HDAC3 protein expression. This attenuation of HDAC3-mediated H3K27 deacetylation at the *CD274* promoter enhances IFN-γ-induced PD-L1 expression and hence facilitates immune evasion in invasive gastric cancer [85]. Similarly, in gastric cancer cells, ZFP36L1 promotes gastric cancer progression by regulating JNK and p38 MAPK signaling pathways, suggesting that combined inhibition of c-Jun N-terminal kinase (JNK) and p38 MAPK could represent a novel therapeutic strategy [123]. In muscle-invasive bladder cancer (MIBC), ZFP36L1 exhibits a dual role: it inhibits the self-renewal and clonal proliferation of bladder cancer cells while activating EMT and transforming growth factor beta (TGF-β) pathways to promote invasion. Consequently, high ZFP36L1 expression is associated with advanced tumor stage, high grade, and poor prognosis in MIBC patients [86].

Together, these findings indicate that ZFP36 family members regulate tumor immunity through multiple immune compartments, including Tregs, effector T cells, macrophages, and immune-evasion pathways. Their immune-microenvironment-dependent effects may be antitumor or pro-tumorigenic depending on the dominant cellular source, target transcript, cytokine network, and disease stage.

### 5.3. Balancing Tumor Suppression and Tumor-Promoting Activity

Accumulating evidence suggests that the ZFP36 family may also exert context-dependent pro-tumorigenic effects in specific tumor types, disease stages, and immune microenvironments [85,123,124,125]. These effects may arise through both tumor-cell-intrinsic mechanisms and immune-microenvironment-dependent regulation. A recent pan-cancer analysis revealed that *ZFP36* is highly expressed in most human tumors. Its expression is associated with poor prognosis in certain malignancies and correlates with tumor-related signaling pathways, immune cell infiltration, and functional genes, such as *SOCS3*, *JUN*, *SLC7A11*, and *CSRNP1*. These findings suggest that ZFP36 may contribute to tumor progression and immune microenvironment remodeling, serving as a critical post-transcriptional regulator [126]. These associations support context dependence but do not by themselves establish direct causality.

Aberrant expression of the ZFP36 family, mediated by non-coding RNAs, constitutes another key mechanism underlying its oncogenic role. In gastric cancer, circ_0003789 competitively binds miR-429, thereby relieving miR-429-mediated inhibition of *ZFP36L2*. This results in significantly elevated *ZFP36L2* expression in gastric cancer tissues and cells, which is negatively correlated with miR-429 levels. Consequently, this upregulation promotes gastric cancer cell proliferation, clonogenicity, and invasiveness while inhibiting apoptosis [89]. In cervical cancer, the elevated *ZFP36L2* expression is closely associated with increased tumor volume and lymph node metastasis, and is significantly negatively correlated with miR-520d-3p expression. miR-520d-3p inhibits *ZFP36L2* expression by binding to its mRNA 3′-UTR; conversely, ZFP36L2 overexpression reverses the inhibitory effects of miR-520d-3p on cervical cancer cell proliferation, migration, and EMT. These findings indicate that ZFP36L2 plays a pro-oncogenic role in cervical cancer and serves as a key mediator of the tumor-suppressive function of miR-520d-3p [88]. In pancreatic ductal adenocarcinoma (PDAC), *ZFP36L2* promotes carcinogenesis by modulating genes associated with the cell cycle and cancer signaling pathways. In contrast, the tumor-suppressor miR-375 directly targets *ZFP36L2* and suppresses its expression, thereby exerting an antitumor effect [87]. Furthermore, in breast cancer, the lncRNA *NNT-AS1* promotes tumor growth by suppressing *ZFP36* expression; knocking down *NNT-AS1* restores the tumor-suppressive effects of ZFP36, which inhibits breast cancer cell proliferation, migration, and tumor growth in a mouse model [127].

Overall, the role of the ZFP36 family in tumors is not strictly tumor-suppressive or tumor-promoting; it depends on specific family members, tumor type, immune microenvironment, and disease stage. Its tumor-suppressive effects are primarily associated with the downregulation of mRNAs related to pro-inflammatory factors, the cell cycle, angiogenesis, EMT, and metabolism. Conversely, its tumor-promoting effects are more commonly observed in the immune evasion, invasion, and metastasis, as well as through the dysfunction of non-coding RNA networks in specific tumor settings.

## 6. ZFP36 Family Proteins in Inflammatory, Autoimmune, and Tissue-Damage Disorders

The persistent expression of inflammatory cytokines and immune effector molecules constitutes a key pathological basis for autoimmune diseases, allergic inflammation, tissue damage, and transplant rejection. The consequences of ZFP36 family activity vary according to disease context, cell type, and immune state. In many chronic inflammatory and autoimmune disorders, ZFP36 family-mediated repression of inflammatory transcripts is protective because it limits sustained cytokine production and immune activation [7,9,16,17,34,128,129]. Given the post-transcriptional regulatory functions described above, the ZFP36 family exerts protective effects in rheumatoid arthritis (RA), experimental autoimmune encephalomyelitis (EAE), acute lung injury (ALI), asthma, allergic rhinitis (AR), and transplant immunity. Consequently, this section systematically reviews the regulatory roles and potential therapeutic value of the ZFP36 family in inflammation, autoimmune diseases, allergic disorders, and transplant immunity.

### 6.1. Chronic Inflammation and Autoimmune Disease

In inflammatory and autoimmune diseases, the protective role of the ZFP36 family is primarily manifested by restricting the persistence of inflammatory responses and preventing the dysregulation of immune effector responses. In chronic inflammatory conditions, such as RA, impaired ZFP36 function leads to persistently elevated levels of inflammatory cytokines, including TNF and IL-6, thereby exacerbating synovial inflammation, joint destruction, and bone erosion [34]. In the EAE model, the ZFP36 family suppresses pro-inflammatory T cell responses and the sustained expression of cytokines, such as IFN-γ, TNF, and granulocyte-macrophage colony-stimulating factor (GM-CSF); its absence induces severe multi-organ inflammatory responses [16]. Concurrently, ZFP36L1 and ZFP36L2 play a crucial role in maintaining the immunosuppressive function of Treg cells. Their knockout alleviates Treg-mediated immune tolerance and promotes the amplification of inflammatory responses [17].

In contrast to chronic diseases, such as RA, ALI is characterized by a rapid escalation of acute inflammatory cascades, accompanied by pulmonary edema, inflammatory cell infiltration, and disruption of the tissue barrier. In ALI models, ZFP36 inhibits excessive macrophage activation and cytokine release, ultimately alleviating pulmonary edema, inflammatory infiltration, and tissue damage [7,9,128].

### 6.2. Allergic Inflammation

In allergic diseases, the ZFP36 family regulates inflammatory responses. Research indicates that ZFP36 family members are suppressed in T helper 2 (Th2) cells in asthma patients, leading to increased secretion of inflammatory cytokines and expression of inflammatory molecules on the cell surface. This upregulation enhances Th2 cell function and exacerbates airway inflammation. Furthermore, cytokines such as IL-2, interleukin-4 (IL-4), and interleukin-15 (IL-15) further suppress their expression, creating a positive feedback loop. Glucocorticoids induce ZFP36 expression, suggesting a potential role for ZFP36 in mediating anti-inflammatory effects. Notably, ZFP36 participates not only in the regulation of Type 2 inflammation but may also influence non-Type 2 inflammation and virus-induced airway inflammatory responses, making it a potential therapeutic target for inflammatory diseases such as asthma [130].

In AR, ZFP36 exerts an immunosuppressive effect by inhibiting Th2 cell activation and differentiation. ZFP36 recognizes and binds to the ARE in the 3′ UTR of the target gene *tripartite motif-containing 18* (*TRIM18*) mRNA, promoting *TRIM18* mRNA degradation. This process reduces TRIM18-mediated Th2 inflammatory signaling, decreases the secretion of Th2 cytokines such as IL-4, IL-5, and IL-13, and inhibits the increase in the proportion of Th2 cells. In contrast, *Zfp36* deficiency exacerbates Th2-biased inflammation, whereas its overexpression significantly alleviates nasal mucosal inflammation and immune cell infiltration. This study demonstrates that ZFP36 maintains T cell immune homeostasis through the ZFP36-TRIM18 axis and serves as a critical post-transcriptional regulator of T cell differentiation and allergic inflammation [131].

### 6.3. Transplant Immunity

Compared with autoimmune and allergic diseases, direct evidence of ZFP36 family function in transplant immunity remains limited and is currently centered on myeloid cell-mediated kidney allograft rejection. Kidney transplantation is considered the optimal treatment for end-stage kidney disease (ESKD). Relevant models have demonstrated that elevated *Zfp36* expression in p21^high^ macrophages promotes degradation of *Il27p28* mRNA and reduces IL-27 production, thereby relieving CD8^+^ T cell activation and cytotoxic responses. Conversely, myeloid-specific *Zfp36* deficiency enhances IL-27 expression and exacerbates acute rejection [90]. Enhancing ZFP36 activity is expected to promote the degradation of inflammation-related mRNAs, such as *Tnf*, *Il6*, and *Il27p28*, ultimately suppressing cytokine expression in transplant rejection or chronic inflammation [7,32,132]. However, given that the ZFP36 family is involved in anti-infective and antitumor immunity as well as the maintenance of Treg homeostasis, therapeutic interventions should not rely on systemic enhancement strategies but should instead focus on targeted regulation within specific cell types and inflammatory pathways [17,73,95]. Importantly, most of the evidence in transplantation immunology comes from murine models of kidney rejection, and direct clinical data are lacking; therefore, the translational relevance of these findings remains to be validated [90].

### 6.4. Neuroinflammatory and Neuroimmune Disorders

Beyond graft rejection itself, accumulating evidence suggests that transplantation-associated systemic immune activation may also influence the central nervous system through neuroimmune interactions [133,134]. Severe peripheral inflammation can increase blood–brain barrier (BBB) permeability, facilitate the infiltration of inflammatory mediators into the brain, and trigger persistent activation of microglia, ultimately contributing to neuroinflammation [133,135]. At present, there is no direct evidence that ZFP36 causes post-transplant psychiatric disorders, post-transplant psychosis, or remission of schizophrenia after cell transplantation. Any potential connection should therefore be framed as indirect and hypothesis-generating, possibly mediated by peripheral inflammation, BBB dysfunction, microglial activation, or neuroimmune signaling rather than by a disease-specific ZFP36 mechanism.

A broader link between ZFP36 and psychiatric or neuroimmune disorders also remains indirect. ZFP36 regulates inflammatory and stress-response programs, and recent Alzheimer’s disease models suggest that ZFP36 suppresses microglial activation by promoting degradation of inflammatory transcripts and inhibiting Z-DNA-binding protein 1 (ZBP1)/NLRP3 inflammasome signaling, thereby reducing IL-1β, interleukin-18 (IL-18), TNF-α, and related inflammatory mediators [136]. Given its central role as a post-transcriptional regulator of inflammatory responses, ZFP36 may therefore represent a molecular link between peripheral immune activation and central neuroinflammation [134,135,136]. Future studies should determine whether modulation of ZFP36 in microglia or infiltrating myeloid cells contributes to the neurological and psychiatric complications associated with transplantation or cell-based immunotherapies.

Together, these findings indicate that ZFP36 family members exert disease- and cell-context-dependent effects across inflammatory, allergic, infectious, tumor, and transplant-related settings. These disease contexts, representative target transcripts, involved cell types, and functional outcomes are summarized in Table 3.

The context-dependent functions summarized in Table 3 provide the rationale for therapeutic strategies aimed at modulating ZFP36 family activity in a cell type-, disease stage-, and target transcript-specific manner.

## 7. Therapeutic Strategies Targeting ZFP36 Family Proteins

The ZFP36 family regulates transcripts encoding inflammatory mediators, immune effector molecules, and tumor-associated factors, making it an attractive but complex therapeutic target. Therapeutic strategies can be grouped into small-molecule modulation, RNA-targeted intervention, and cell-specific delivery. However, the current translational status differs substantially across strategies: most direct evidence is experimental or preclinical, especially for ZFP36 restoration, phosphorylation modulation, or RNA-level perturbation, whereas clinically feasible approaches remain hypothetical. Current evidence is strongest for ZFP36-directed modulation in inflammatory and tumor models; ZFP36L1 and ZFP36L2 are more closely linked to lymphocyte quiescence, Treg function, tumor immunity, and non-coding RNA networks. Future interventions must therefore be tailored to disease stage, cell type, and family members. Safety evaluation should explicitly consider systemic inflammation, impaired Treg stability, altered antitumor immunity, impaired host defense, and unintended effects on tissue repair.

### 7.1. Small-Molecule Modulation

Small-molecule interventions primarily aim to restore mRNA degradation function or reverse functional inhibition in pathological conditions. ZFP36 is phosphorylated by various kinases, including ERK, p38 MAPK, JNK, and protein kinase B (AKT); this phosphorylation status affects protein stability and mRNA degradation activity [137]. Persistent p38 MAPK-MK2 signaling can transiently inhibit ZFP36 anti-inflammatory activity by promoting phosphorylation and 14-3-3 binding. Coronavirus infection, for example, activates the mitogen-activated protein kinase kinase 3 (MKK3)-p38-MK2-ZFP36 axis and affects ARE-containing inflammatory transcript regulation [138]. These observations support pathway-level modulation but have not yet established a selective ZFP36-directed drug strategy.

In tumors, small-molecule compounds can also restore the tumor-suppressing function of *ZFP36* by reversing its transcriptional silencing. High methylation of the *ZFP36* promoter in NSCLC results in *ZFP36* epigenetic silencing. Resveratrol directly reduces cytosine-phosphate-guanine (CpG) island methylation in the *ZFP36* promoter by downregulating DNA methyltransferase 1 (DNMT1), thereby relieving transcriptional repression and significantly upregulating *ZFP36* mRNA and protein expression. Upon restoration of *ZFP36* expression, the protein binds AREs in the 3′ UTRs of target genes, such as *CCND1*, *MYC*, and *vascular endothelial growth factor A* (*VEGFA*), promoting their mRNA degradation and ultimately suppressing malignant phenotypes, including proliferation and migration in lung cancer cells [118]. Additionally, in squamous cell carcinoma, abnormally activated Wnt/β-catenin signaling induces EMT-associated transcriptional repressors, such as SNAI1, SLUG, and TWIST; blocking this pathway could alleviate repression and restore *ZFP36* expression [139].

These studies suggest that *ZFP36* expression and activation can be indirectly restored through epigenetic regulation, inhibition of oncogenic signaling, and modulation of phosphorylation. Small-molecule strategies, including resveratrol, Wnt/β-catenin pathway inhibitors, and MK2-related inhibitors, may provide a pharmacological basis for restoring ZFP36-mediated mRNA degradation. However, no specific drugs currently target ZFP36 directly, and its complex post-translational regulatory mechanisms and broad biological functions pose significant challenges for drug development.

Current evidence for small-molecule modulation is primarily derived from cellular and animal studies, and further validation of efficacy and safety is required before clinical application.

### 7.2. RNA-Targeted Approaches

In recent years, RNA-based strategies targeting the expression and function of the ZFP36 family have attracted increasing attention. Among these, microRNAs (miRNAs) regulate breast cancer progression by directly binding to *ZFP36* mRNA and suppressing its expression, whereas certain lncRNAs and circRNAs indirectly restore ZFP36 levels through the ceRNA mechanism [111,127].

Furthermore, the negative regulatory role of ZFP36 in maintaining immune homeostasis and limiting inflammatory responses provides a theoretical basis for targeted RNA-level interventions. Studies have shown that inhibiting *ZFP36* expression with small interfering RNA (siRNA) or short hairpin RNA (shRNA) enhances T cell activation, proliferation, and effector molecule expression, thereby boosting antiviral immune responses in specific infections or immune responses [73]. In macrophage and inflammation models, downregulation of *ZFP36* increases the expression of inflammatory factors, such as TNF-α, ultimately amplifying the innate immune response [57]. These findings, derived mainly from in vitro and murine systems, suggest that transient RNA interference against *ZFP36* could be explored as an immunostimulatory strategy, for example, in vaccine-adjuvant or anti-infective settings. However, the same approach could provoke excessive inflammation, autoimmunity, cytokine toxicity, or tissue damage if delivered systemically or persistently.

Morpholino antisense oligonucleotides have been used experimentally to block mRNAs encoding ZFP36 family members during Xenopus development, inducing anterior kidney abnormalities and demonstrating technical targetability at the RNA level [140]. In contrast, antisense oligonucleotides (ASOs), siRNAs, and miRNA mimics or inhibitors offer more clinically mature RNA-therapeutic platforms in principle [141], but ZFP36 family applications remain largely preclinical. Major unresolved issues include delivery efficiency, cell specificity, dosing window, duration of modulation, off-target transcript effects, and consequences for Treg stability, host defense, and antitumor immunity.

RNA-based approaches remain largely at the preclinical stage and require further optimization of delivery efficiency and tissue specificity. In addition, systemic modulation of ZFP36 activity may increase the risk of systemic inflammation or impair protective immune responses, highlighting the importance of cell-specific delivery strategies.

### 7.3. Cell-Specific Intervention Strategies

Although suppression or knockout of *ZFP36* enhances T cell activation, proliferation, and cytokine expression in certain inflammatory and viral models [73], the efficacy of this strategy in engineered T cells remains limited [91]. In human Mesothelin-targeted Chimeric Antigen Receptor (Meso CAR)-T cells, *ZFP36* knockout alone only mildly enhanced non-antigen-dependent activation and cytokine secretion in CD4^+^ CAR-T cells, without significantly improving antigen-specific killing, proliferation, or antitumor efficacy [91]. These findings suggest that functional redundancy within the ZFP36 family; accordingly, knocking out a single member is insufficient to enhance CAR-T cell function, and combined knockouts of multiple family members or co-regulation with other immune regulatory factors are required for optimal efficacy [91]. Furthermore, the role of the ZFP36 family in Treg stability indicates that its regulation may influence the immunosuppressive tumor microenvironment, hence indirectly enhancing effector T cell function [17]. As an RNA-binding protein that restrains effector T-cell responses, *ZFP36* holds significant potential in combination immunotherapy strategies, such as synergistic regulation with immune checkpoint inhibitors or other RNA-binding proteins. However, current evidence is limited to preclinical CAR-T and mouse tumor models, and clinical applicability has not been established.

In myeloid cells, ZFP36 limits sustained inflammatory-factor expression and may reduce excessive inflammation in sepsis, rheumatoid arthritis, inflammatory bowel disease, tissue injury, and transplant rejection models [9,32,34,128,129,142]. Beyond classical cytokines and chemokines, ZFP36 also influences metabolic pathways [18] and macrophage polarization [122], highlighting myeloid-specific regulation of ZFP36 and its signaling as a promising post-transcriptional strategy for treating inflammatory diseases.

Compared with systemic intervention, cell-specific targeting may provide greater therapeutic precision while minimizing off-target effects. Nevertheless, manipulation of ZFP36 family proteins carries substantial risks. Enhancing effector T cell activity by inhibiting ZFP36 family restraints could improve anti-viral or antitumor responses but may increase immune-mediated tissue damage. Conversely, excessive inhibition in Tregs may compromise Treg stability and precipitate autoimmune inflammation, whereas excessive enhancement in effector or myeloid compartments could suppress host defense or antitumor immunity. These risks should be incorporated into the design of any future therapeutic strategy rather than mentioned only as theoretical caveats. The principal therapeutic strategies, potential applications, and current limitations of targeting the ZFP36 family are summarized in Table 4.

## 8. Future Directions and Outstanding Questions

Although a systematic understanding of the ZFP36 family’s role in post-transcriptional regulation has emerged, several questions regarding its functions in immune regulation and disease remain to be clarified. First, the functional differences among ZFP36 family members across immune cell types and within the tissue microenvironment require further analysis. Existing studies indicate that ZFP36 primarily limits cytokine expression in macrophages, whereas in T cells, it regulates activation, differentiation, and immune tolerance; however, the functional distinctions and dynamic regulatory mechanisms across disease stages remain unclear [10,73]. Additionally, while ARE-dependent mRNA degradation mediated by the ZFP36 family is widely accepted, high-throughput crosslinking and immunoprecipitation sequencing (CLIP-seq) studies suggest that ZFP36 may bind to a broader range of RNA sequences or structural elements. Consequently, the existence of ARE-independent target recognition patterns remains debated [55]. Furthermore, the mechanisms underlying competition or synergy between ZFP36 and other RNA-binding proteins, such as HuR, need to be elucidated; clarifying these interactions may be the key to explaining their functional differences in inflammation and tumors [143]. Regarding functional regulation, further research is needed on the post-translational modifications and subcellular localization of ZFP36. Phosphorylation mediated by the p38 MAPK-MK2 pathway is considered critical for regulating its function; however, systematic studies on the interrelationships among different modifications and their dynamic changes across various disease states await further investigation [138,143]. Meanwhile, how spatial localization within RNA granules (such as PBs and TIS granules) influences mRNA fate remains a key area of current research. Finally, although the ZFP36 family exhibits dual functions in immune diseases and tumors, the specific regulatory mechanisms by which it modulates different immune cells, such as Tregs, γδ T cells, and myeloid cells, in the tumor immune microenvironment have not yet been elucidated [73]. Although RNA interference technologies, including siRNA and ASO, have progressed in treating other diseases [141], RNA-targeted therapies for the ZFP36 family remain in the theoretical exploration phase. Future research must identify the optimal intervention window for regulating its expression and elucidate the differences in safety and efficacy between direct targeting and indirect regulatory strategies. Future therapeutic development should prioritize precise modulation according to the targeted cell type, disease stage, and specific ZFP36 family member to maximize efficacy while minimizing immune-related adverse effects.

In summary, future research should focus on the cell-type-specific functions of the ZFP36 family, non-canonical RNA recognition mechanisms, post-translational regulatory networks, and potential immunotherapy applications. Addressing these issues will facilitate the translation of the ZFP36 family from basic research to targeted therapeutic applications.

## 9. Conclusions

In conclusion, the ZFP36 family represents a post-transcriptional regulatory system with checkpoint-like properties, because it limits excessive immune activation through RNA-mediated control of immune effector programs in a manner distinct from canonical receptor-based immune checkpoints. This designation should be used precisely: ZFP36 family proteins are intracellular RNA-binding regulators, not classical immune checkpoint receptors. Through ARE-dependent mRNA decay and translational repression, they regulate the magnitude and duration of inflammatory and immune responses across diverse cell types. ZFP36 often acts as an inducible negative-feedback regulator in myeloid and epithelial inflammatory contexts, whereas ZFP36L1 and ZFP36L2 are especially important for lymphocyte quiescence, T cell fitness, Treg stability, and B cell development. In cancer, their effects are context-dependent and should be divided into tumor-cell-intrinsic and immune-microenvironment-dependent mechanisms. Therapeutically, small molecules, RNA-based approaches, and cell-specific interventions remain promising but largely experimental or pre-clinical. Future research should prioritize direct target validation, family-member-specific mechanisms, disease-stage-specific functions, and precision delivery systems that maximize benefit while minimizing systemic inflammation, impaired Treg stability, altered antitumor immunity, and compromised host defense.

## Figures and Tables

**Table 1:** Human ZFP36 family RNA-binding proteins.

Gene Name	Aliases	Ref-Seq Protein	Amino Acids	MW (kDa)
*ZFP36*	TTP, TIS11	NP_003398	326	34.0
*ZFP36L1*	TIS11B, BRF1	NP_004917	338	36.3
*ZFP36L2*	TIS11D, BRF2	NP_008818	494	51.0

**Table 2:** Comparative summary of experimentally validated RNA targets and inferred regulatory mechanisms of ZFP36 family members across immune and disease contexts.

Member(s)	Cell Type/Context	Target or Pathway	Evidence Category/Strength	Functional Implication	Refs.
ZFP36	Macrophages; acute inflammation	Validated target: *Tnf*	Direct target validation; Strong (ARE-dependent decay and genetic evidence)	ZFP36 limits TNF-α-driven systemic inflammation.	[32,34]
ZFP36	Bone marrow-derived dendritic cells; inflammatory amplification	Supported target: *Il23*	Target-level regulation; Strong (mRNA-stability and disease-model evidence)	ZFP36 restrains IL-23-mediated inflammatory amplification.	[58]
ZFP36	Neutrophils; bacterial infection	Validated target: *Mcl1*	Direct target validation; Strong (mRNA-destabilization and myeloid genetic evidence)	ZFP36 promotes apoptosis of activated neutrophils during infection resolution.	[59]
*Zfp36*, *Zfp36l1*, *Zfp36l2* (combined deletion)	Myeloid cells; systemic inflammatory disease	Transcript program: cytokine and chemokine transcripts; Transcript-program level; Member-specific targets incompletely mapped	Combined genetic evidence; Strong (combined conditional deletion evidence)	Combined deletion demonstrates cooperative regulation of myeloid inflammatory responses.	[20]
ZFP36	ILC2s; type 2 immunity	Validated target: *Il5*; Indirect pathway: IL-13 production	Direct target validation for Il5; Pathway-level for IL-13; Strong/moderate	ZFP36 restrains constitutive type 2 cytokine production.	[62]
ZFP36	Intestinal ILC3s; DSS-induced colitis	Pathway: IL-22-related response; Direct target unresolved	Pathway-level evidence; Moderate (Disease-model evidence with unresolved direct target)	ZFP36 modulates IL-22-associated intestinal inflammation.	[63,67,68]
ZFP36	Keratinocytes; skin inflammation and carcinogenesis	Validated target: *Areg*; transcript program: Inflammatory cytokine/chemokine network	Direct target validated for *Areg*; Strong for *Areg*; Pathway/transcript-program level for broader network; Strong/moderate to strong	ZFP36 limits keratinocyte-driven inflammatory and tumor-promoting signals.	[67,68,70]
ZFP36	Airway epithelial cells; cystic fibrosis inflammation	Supported target: *CXCL8* mRNA/IL-8 protein	Target-level validation; Moderate to strong (epithelial mRNA-stability evidence)	ZFP36 promotes *CXCL8* mRNA decay in airway epithelium.	[70,71]
ZFP36L1, ZFP36L2	Airway epithelium; severe asthma	Inferred targets: predicted inflammatory transcripts	Inferred/correlative evidence; Limited to moderate (expression-association evidence; Direct validation required)	ZFP36L1 and ZFP36L2 are associated with severe asthma epithelial dysregulation.	[71,73]
ZFP36	Activated CD4^+^ and CD8^+^ T cells; antiviral immunity	Validated targets: *Cd69*, *Il2*, *Ifng*, *Tnf*;	Direct target validation; Strong (transcript-level and in vivo functional evidence)	ZFP36 restrains T cell activation and antiviral effector expansion.	[16,73]
*Zfp36*, *Zfp36l1*, *Zfp36l2* (combined deletion)	CD4^+^ and CD8^+^ T cells; homeostasis and autoreactivity	Transcript program: activation, quiescence, and effector transcripts; Member-specific targets incompletely mapped	Combined genetic evidence; Strong (combined conditional deletion with transcript-program effects)	The family members cooperatively maintain T cell quiescence and immune tolerance.	[16,74]
*Zfp36*, *Zfp36l1* (dual deletion)	CD8^+^ T cells; influenza infection	Transcript program: effector differentiation transcripts; Activation-state-dependent direct targets	Combined genetic evidence; Strong (dual-deletion functional evidence)	ZFP36 and ZFP36L1 cooperatively delay CD8^+^ effector differentiation.	[74,75]
ZFP36L1	CD8^+^ T cells; antigen-affinity-dependent response	Pathway: IL-2 responsiveness/TCR affinity signaling; Direct targets require validation	Pathway-level mechanism; Moderate to strong (signaling and functional evidence)	ZFP36L1 links TCR affinity to cytokine responsiveness.	[33,75]
ZFP36L2	Activated T cells; late effector response	Validated target: *Ifng*	Direct target validation; Strong (ARE-dependent mRNA-destabilization evidence)	ZFP36L2 limits late-phase IFN-γ production.	[33]
*Zfp36l1*, *Zfp36l2*(double deletion)	T cells; cytokine production and fitness	Transcript program: cytokine and cell-cycle transcripts; Direct targets incompletely mapped	Combined genetic evidence; Strong (double-deletion functional evidence)	ZFP36L1 and ZFP36L2 balance cytokine output with T cell fitness.	[76]
*Zfp36l1*, *Zfp36l2*(Treg-specific double deletion)	Regulatory T cells; immune tolerance	Pathway: IFN-γ response, CTLA-4 recycling, and IL-2/IL-7 responsiveness; Direct targets incompletely defined	Combined genetic plus pathway-level evidence; Strong (Treg-specific double-deletion evidence)	ZFP36L1 and ZFP36L2 maintain Treg stability and suppressive function.	[17]
ZFP36L2	Inducible Treg differentiation	Validated target: *Ikzf2*/Helios	Direct target validations; Strong (target-level and functional evidence)	ZFP36L2 modulates Helios expression and iTreg suppressive function.	[77]
*Zfp36l1*, *Zfp36l2*(double deletion)	Developing B cells; B cell development	Transcript program: cell-cycle-related transcripts; Individual targets incompletely resolved	Combined genetic evidence; Strong (genetic and developmental evidence)	ZFP36L1 and ZFP36L2 maintain precursor B cell quiescence.	[19]
ZFP36L1	Marginal zone B cells; mature B cell homeostasis	Transcript program: signaling, Adhesion, and locomotion transcripts; direct targets partially resolved	Pathway-level evidence; Strong functional evidence; moderate target resolution	ZFP36L1 supports marginal zone B cell maintenance and localization.	[78]
ZFP36L1	Antibody-secreting cells; plasma cell homing	Transcript program: migration and homing transcripts; direct targets incompletely resolved	Pathway-level evidence; Moderate to strong (B cell functional evidence)	ZFP36L1 promotes antibody-secreting cell homing to bone marrow.	[79]
*Zfp36l1*, *Zfp36l2*(double deletion)	Germinal center B cells; humoral immunity	Transcript program: cell-cycle and replication-stress transcripts; Direct targets still emerging	Combined genetic/pathway-level evidence; Moderate to strong (checkpoint-related functional evidence)	ZFP36L1 and ZFP36L2 protect germinal center B cells from replication stress.	[80]
ZFP36L1	Influenza A virus-infected cells	Validated viral RNA targets: HA, M, and NS segments	Direct target validation; Strong (viral RNA targeting and translational repression evidence)	ZFP36L1 suppresses influenza viral protein production.	[81]
ZFP36L1	Flavivirus-infected cells	Pathway: XRN1- and exosome-mediated viral RNA decay	Pathway-level evidence; Strong (viral RNA-decay evidence)	ZFP36L1 restricts flavivirus infection through RNA decay pathways.	[82]
ZFP36	NSCLC cells; chemoresistance	Validated target: *FASN*	Direct target validation; Strong (ARE-dependent decay and tumor functional evidence)	ZFP36 suppresses lipid-droplet-associated chemoresistance.	[83]
ZFP36L1	Liver cancer cells; EMT and metastasis	Validated target: *ZEB2*	Direct target validation; Strong (3′ UTR/ARE-associated regulation and tumor functional evidence)	ZFP36L1 suppresses EMT and metastatic potential.	[84]
ZFP36L1	Gastric cancer cells; immune evasion	Validated target: *HDAC3* mRNA; Pathway: PD-L1 regulation	Direct target validation for *HDAC3* plus pathway-level evidence for PD-L1; Moderate to strong	ZFP36L1 promotes IFN-γ-induced PD-L1 expression in gastric cancer.	[85]
ZFP36L1	Muscle-invasive bladder cancer	Pathway: EMT and TGF-β signaling; Direct targets not fully defined	Pathway-level evidence; Moderate (tumor functional evidence)	ZFP36L1 shows context-dependent effects on self-renewal and invasion.	[86]
ZFP36L2	Gastric cancer, cervical cancer, and PDAC models	Pathway: miRNA/circRNA–*ZFP36L2* regulatory axes; Direct target validation varies	Pathway-level evidence; Moderate (con-text-dependent tumor functional evidence)	ZFP36L2 can promote tumor aggressiveness in selected contexts.	[87,88,89]
ZFP36	p21^high^ macrophages and CD8^+^ T cells; kidney transplant rejection	Supported target: *Il27p28*; Pathway: IL-27 axis	Direct/target-level validation plus pathway-level evidence; Strong (preclinical transplant and mRNA-degradation evidence)	ZFP36 suppresses IL-27-linked CD8^+^ T cell cytotoxicity in renal allografts.	[90]
ZFP36	Mesothelin-targeted Chimeric Antigen Receptor (Meso CAR)-T cells	Transcript program: cytokine and activation transcripts; No beneficial mechanism established	Perturbation/pathway-level evidence; Preclinical negative evidence	*ZFP36* disruption alone is insufficient to enhance CAR-T cell efficacy.	[91]

Note: The terms used in the “Target or pathway” and “Evidence category/strength” columns indicate the type and directness of mechanistic evidence. “Validated target” denotes direct experimental evidence for transcript-specific RNA regulation, whereas “supported target” denotes transcript-specific evidence with less complete mechanistic validation. “Transcript program,” “pathway,” and “inferred targets” indicate broader or less direct levels of evidence in which individual RNA targets remain incompletely defined. Rows listing multiple ZFP36 family members generally reflect combined or comparative perturbation studies and should not be interpreted as evidence that each member regulates identical direct target mRNAs.

**Table 3:** Disease contexts, target transcripts, involved cell types, and functional outcomes of ZFP36 family members.

Disease/Biological Context	Family Member(s)	Major Cell Type(s)	Validated Targets or Representative Regulated Transcripts	Functional Outcome	Refs.
Inflammatory arthritis/RA-like inflammation	ZFP36	Macrophages and inflammatory myeloid cells	*Tnf*; *Il6*	Restrains inflammatory cytokine production and joint inflammation.	[32,34,129,132]
Multiple sclerosis/EAE-related inflammation	ZFP36L1, ZFP36L2	Regulatory T cells	*Ifng*-associated program; direct targets not fully defined	Maintains Treg suppressive function and immune tolerance.	[16,17,73]
Myeloid inflammation	ZFP36, ZFP36L1, ZFP36L2	Myeloid cells	Multiple inflammatory transcripts	Limits systemic myeloid inflammatory amplification.	[20]
Psoriasis-like skin inflammation	ZFP36	Keratinocytes	*Tnf*; *Cxcl1*; *Cxcl2*	Limits keratinocyte-driven skin inflammation.	[67]
Cystic fibrosis airway inflammation	ZFP36	Airway epithelial cells	*CXCL8* mRNA/IL-8 protein	Promotes *CXCL8* mRNA decay and limits neutrophilic airway inflammation.	[70]
Intestinal inflammation/DSS colitis	ZFP36	Intestinal epithelial cells and ILC3s	*Nos2*; IL-22-related target not fully defined	Modulates epithelial and ILC3-associated colitis susceptibility.	[63,72]
Bacterial infection	ZFP36	Neutrophils	*Mcl1*	Promotes apoptosis of pathogen-engaged neutrophils and inflammatory resolution.	[59]
Asthma/type 2 inflammation	ZFP36	ILC2s	*Il5*; *Il13* indirectly regulated	Suppresses constitutive type 2 cytokine production.	[62]
Severe asthma	ZFP36L1, ZFP36L2	Airway epithelial cells	Not fully defined; predicted inflammatory transcripts	Associated with epithelial inflammatory dysregulation; direct targets remain to be validated.	[71]
Allergic rhinitis	ZFP36	Th2 cells	*TRIM18*	Suppresses Th2 activation and allergic inflammation.	[72,131]
Kidney transplant rejection	ZFP36	p21^high^ macrophages and CD8^+^ T cells	*Il27p28*	Protects renal allografts by limiting IL-27-linked CD8^+^ T-cell cytotoxicity.	[90]
Alzheimer’s disease	ZFP36	Microglia	Inflammatory mediators and NLRP3 inflammasome signaling	Inhibiting microglia activation and relieving nerve inflammation	[136]
Antiviral T-cell immunity	ZFP36, ZFP36L1	CD4^+^ and CD8^+^ T cells	*Cd69*; *Il2*; *Ifng*; *Tnf*	Restrains T-cell activation and antiviral effector expansion.	[73,74,131]
Influenza virus infection	ZFP36L1	Virus-infected cells	HA; M; NS viral RNAs	Restricts influenza virus replication by suppressing viral protein production.	[81]
Flavivirus infection	ZFP36L1	Virus-infected cells	Viral RNA; specific targets not fully defined	Promotes XRN1- and exosome-mediated viral RNA decay.	[82]
B-cell development and humoral immunity	ZFP36L1, ZFP36L2	Developing and germinal-center B cells	Cell-cycle-associated transcripts	Maintains B-cell quiescence and protects proliferating B cells from replication stress.	[19,74,78,79,80]
Skin carcinogenesis	ZFP36	Keratinocytes	*Areg*	Suppresses EGFR-associated tumor-promoting inflammation.	[68]
Cancer cell biology	ZFP36, ZFP36L1, ZFP36L2	Tumor cells	*FASN*; *ZEB2*; *HDAC3*; ZFP36L2-associated targets not fully defined	Modulates chemoresistance, EMT, invasion, and tumor progression.	[83,84,85,86,87,88,89]
Antitumor immunity	ZFP36 family	CD8^+^ T cells, macrophages, and Tregs	*Ifng*; *Tnf*; *Il2*; *Il27p28*	Restrains immune effector programs but may compromise antitumor immunity.	[17,73,95,122]

Note: Representative targets include validated transcripts or the most relevant transcripts discussed in the cited studies. “Not fully defined” indicates that the functional phenotype has been reported, but direct RNA targets remain incompletely validated. Entries listing multiple family members generally reflect combined or comparative studies and do not imply identical direct RNA targets for each member.

**Table 4:** Therapeutic strategies and intervention potential based on the ZFP36 family.

Therapeutic Category	Representative Strategy	Targeted Mechanism	Potential Application	Current Limitations	Refs.
Small-molecule modulation	MK2/p38 inhibitor	Restore ZFP36 activity by preventing inhibitory phosphorylation	Autoimmune diseases and chronic inflammation	Lack of highly selective ZFP36-directed inhibitors; pathway overlap	[118,137,138,139]
	Resveratrol	Upregulate *ZFP36* through epigenetic regulation	Inflammatory disorders and cancer	Limited bioavailability and indirect mechanism	
	Wnt/β-catenin inhibitor	Restoring *ZFP36* expressions by relieving transcriptional repression	Cancer therapy	Mostly preclinical evidence; broad pathway effects	
RNA-based approaches	miRNA	Regulate *ZFP36* family expression	Precision RNA therapeutics	Mostly preclinical evidence; broad pathway effects	[57,73,111,127,140,141]
	lncRNA/circRNA	Modulate ceRNA networks controlling *ZFP36* family expression	Tumor intervention	Mechanistic complexity and context dependence	
	siRNA/shRNA	Knock down selected *ZFP36* family genes	Functional studies or transient immune enhancement	Off-target effects and risk of excessive inflammation	
	Morpholino ASO	Block *ZFP36* family expression	Experimental validation of family-member function	Limited clinical translation	
Cell-specific intervention	CAR-T engineering	Manipulate ZFP36 family activity to enhance T-cell function	Adoptive immunotherapy	Single-gene disruption may be insufficient; risk of excessive immune activation	[9,17,18,32,34,73,91,122,128,129,132,142,143]
	Treg modulation	Regulate ZFP36L1/ZFP36L2-dependent Treg stability and suppressive function	Autoimmune disease or tumor immune modulation	Requires precise cell-specific delivery	
	Myeloid/macrophage targeting	Control inflammatory transcript turnover and inflammatory amplification	Chronic inflammatory diseases and tissue injury	Targeting efficiency and macrophage-subset specificity	

## Data Availability

No new data were created or analyzed in this study. Data sharing is not applicable.

## Abbreviations

The following abbreviations are used in this manuscript:

3′ UTRs	3′ untranslated regions
4EHP	eIF4E-homologous protein
4E-T	eIF4E transporter
AKT	protein kinase B
ALI	Acute lung injury
AR	Allergic rhinitis
AREs	AU-rich elements
ASOs	Antisense oligonucleotides
BMDCs	Marrow-derived dendritic cells
BRF	Butyrate Response Factor
CCND1	Cyclin D1
CCR4-NOT	Carbon catabolite repression 4-Negative on TATA-less
cDC2s	conventional type 2 dendritic cells
ceRNA	competing endogenous RNA
circRNA	Circular RNA
CLIP-seq	crosslinking and immunoprecipitation sequencing
CNOT	CCR4-NOT transcription complex subunit 1
CpG	cytosine-phosphate-guanine
CSRNP1	Cysteine-serine-rich nuclear protein-1
CTLA-4	Cytotoxic T-lymphocyte-associated protein 4
CTLs	Cytotoxic T lymphocytes
CXCL8	C-X-C motif chemokine ligand 8
DDX6	DEAD-box helicase 6
DNMT1	DNA methyltransferase 1
DSS	dextran sulfate sodium
EAE	Experimental autoimmune encephalomyelitis
EGFR	Epidermal growth factor receptor
eIF4E2	eukaryotic translation initiation factor 4E family member 2
EMT	Epithelial–mesenchymal transition
ERK	extracellular signal-regulated kinase
ESKD	End-stage kidney disease
EZH2	Enhancer of Zeste homolog 2
FASN	fatty acid synthase
FOXP3	forkhead box P3
GM-CSF	Granulocyte-macrophage colony-stimulating factor
GYF2	GRB10-interacting GYF protein 2
HA	hemagglutinin
HDAC3	Histone deacetylase 3
HuR	Human antigen R
IKZF2	Ikaros zinc finger 2
IL-6	interleukin-6
ILCs	Innate lymphoid cells
iTreg	inducible regulatory T cell
JNK	c-Jun N-terminal kinase
LCMV	Lymphocytic choriomeningitis virus
LD	Lipid droplet
LPS	Lipopolysaccharide
lncRNA	long non-coding RNA
M	matrix
m^7^G	7-methylguanosine
Mcl1	Myeloid cell leukemia 1
Meso CAR-T	Mesothelin-targeted Chimeric Antigen Receptor-T
MIBC	Muscle-invasive bladder cancer
MK2	MAP kinase-activated protein kinase 2
MKK3	mitogen-activated protein kinase kinase 3
MZ B cells	Marginal zone B cells
NES	Nuclear export signal
NF-κB	Nuclear factor-kappa B
NLRP3	NOD-, LRR- and pyrin domain-containing protein 3
NLS	Nuclear localization signal
NS	nonstructural
NSCLC	Non-small cell lung cancer
PABPC1	Poly(A)-binding protein cytoplasmic 1
P-bodies	Processing bodies
PD-1	programmed death 1
PDAC	Pancreatic ductal adenocarcinoma
PD-L1	programmed death-ligand 1
PKR	protein kinase R
RA	Rheumatoid arthritis
RBPs	RNA-binding proteins
SEs	Super-enhancers
SGs	Stress granules
shRNA	short hairpin RNA
siRNA	small interfering RNA
SKI	superkiller
SLC7A11	Solute carrier family 7 member 11
SOCS3	Suppressor of cytokine signaling 3
STAT3	Signal transducer and activator of transcription 3
TCR	T cell receptor
Tfh	T follicular helper
TGF-β	transforming growth factor beta
Th2	T helper 2
TIS	TPA-inducible sequence
TNF-α	Tumor necrosis factor alpha
Tregs	regulatory T cells
TRIM18	tripartite motif-containing 18
TTP	Tristetraprolin
TZF	Tandem zinc finger
UPR	Unfolded protein response
VEGF	Vascular endothelial growth factor
VEGFA	vascular endothelial growth factor A
XRN1	Exoribonuclease 1
ZBP1	Z-DNA-binding protein 1
ZFP36	Zinc finger protein 36
ZFP36L1	ZFP36 CCCH-type like 1
ZFP36L2	ZFP36 CCCH-type like 2
ZnF	zinc finger

## References

[1] Turner M., Petkau G. (2026). RNA-binding proteins and ribonucleoproteins as determinants of immunity. Nat. Rev. Immunol..

[2] Akiyama T., Suzuki T., Yamamoto T. (2021). RNA decay machinery safeguards immune cell development and immunological responses. Trends Immunol..

[3] Panthi A., Lynch K.W. (2025). RNA processing in innate immunity: Regulation by RNA-binding proteins. Trends Biochem. Sci..

[4] He S., Valkov E., Cheloufi S., Murn J. (2023). The nexus between RNA-binding proteins and their effectors. Nat. Rev. Genet..

[5] Bestehorn A., von Wirén J., Zeiler C., Fesselet J., Didusch S., Forte M., Doppelmayer K., Borroni M., Le Heron A., Scinicariello S. (2025). Cytoplasmic mRNA decay controlling inflammatory gene expression is determined by pre-mRNA fate decision. Mol. Cell.

[6] Lee T.A., Han H., Polash A., Cho S.K., Lee J.W., Ra E.A., Lee E., Park A., Kang S., Choi J.L. (2022). The nucleolus is the site for inflammatory RNA decay during infection. Nat. Commun..

[7] Snyder B.L., Blackshear P.J. (2022). Clinical implications of tristetraprolin (TTP) modulation in the treatment of inflammatory diseases. Pharmacol. Ther..

[8] Pekovic F., Lai W.S., Corbo J., Hicks S.N., Luke K., Blackshear P.J., Valkov E. (2025). Multivalent interactions with CCR4-NOT and PABPC1 determine mRNA repression efficiency by tristetraprolin. Nat. Commun..

[9] Makita S., Takatori H., Nakajima H. (2021). Post-Transcriptional Regulation of Immune Responses and Inflammatory Diseases by RNA-Binding ZFP36 Family Proteins. Front. Immunol..

[10] Brooks S.A., Blackshear P.J. (2013). Tristetraprolin (TTP): Interactions with mRNA and proteins, and current thoughts on mechanisms of action. Biochim. Biophys. Acta.

[11] Buchbinder E.I., Desai A. (2016). CTLA-4 and PD-1 Pathways: Similarities, Differences, and Implications of Their Inhibition. Am. J. Clin. Oncol..

[12] Stoecklin G., Stubbs T., Kedersha N., Wax S., Rigby W.F., Blackwell T.K., Anderson P. (2004). MK2-induced tristetraprolin:14-3-3 complexes prevent stress granule association and ARE-mRNA decay. EMBO J..

[13] Chrestensen C.A., Schroeder M.J., Shabanowitz J., Hunt D.F., Pelo J.W., Worthington M.T., Sturgill T.W. (2004). MAPKAP kinase 2 phosphorylates tristetraprolin on in vivo sites including Ser178, a site required for 14-3-3 binding. J. Biol. Chem..

[14] Rezcallah M.C., Al-Mazi T., Ammit A.J. (2021). Cataloguing the phosphorylation sites of tristetraprolin (TTP): Functional implications for inflammatory diseases. Cell. Signal..

[15] Scinicariello S., Soderholm A., Schäfer M., Shulkina A., Schwartz I., Hacker K., Gogova R., Kalis R., Froussios K., Budroni V. (2023). HUWE1 controls tristetraprolin proteasomal degradation by regulating its phosphorylation. eLife.

[16] Cook M.E., Bradstreet T.R., Webber A.M., Kim J., Santeford A., Harris K.M., Murphy M.K., Tran J., Abdalla N.M., Schwarzkopf E.A. (2022). The ZFP36 family of RNA binding proteins regulates homeostatic and autoreactive T cell responses. Sci. Immunol..

[17] Sáenz-Narciso B., Bell S.E., Matheson L.S., Venigalla R.K.C., Turner M. (2025). ZFP36-family RNA-binding proteins in regulatory T cells reinforce immune homeostasis. Nat. Commun..

[18] Turner M., Díaz-Muñoz M.D. (2018). RNA-binding proteins control gene expression and cell fate in the immune system. Nat. Immunol..

[19] Galloway A., Saveliev A., Łukasiak S., Hodson D.J., Bolland D., Balmanno K., Ahlfors H., Monzón-Casanova E., Mannurita S.C., Bell L.S. (2016). RNA-binding proteins ZFP36L1 and ZFP36L2 promote cell quiescence. Science.

[20] Snyder B.L., Huang R., Burkholder A.B., Donahue D.R., Mahler B.W., Bortner C.D., Lai W.S., Blackshear P.J. (2024). Synergistic roles of tristetraprolin family members in myeloid cells in the control of inflammation. Life Sci. Alliance.

[21] Blackshear P.J., Perera L. (2014). Phylogenetic distribution and evolution of the linked RNA-binding and NOT1-binding domains in the tristetraprolin family of tandem CCCH zinc finger proteins. J. Interferon Cytokine Res..

[22] Blackshear P.J., Phillips R.S., Ghosh S., Ramos S.V.B., Richfield E.K., Lai W.S. (2005). Zfp36l3, a Rodent X Chromosome Gene Encoding a Placenta-Specific Member of the Tristetraprolin Family of CCCH Tandem Zinc Finger Proteins. Biol. Reprod..

[23] Stumpo D.J., Trempus C.S., Tucker C.J., Huang W., Li L., Kluckman K., Bortner D.M., Blackshear P.J. (2016). Deficiency of the placenta- and yolk sac-specific tristetraprolin family member ZFP36L3 identifies likely mRNA targets and an unexpected link to placental iron metabolism. Development.

[24] Gingerich T.J., Stumpo D.J., Lai W.S., Randall T.A., Steppan S.J., Blackshear P.J. (2016). Emergence and evolution of Zfp36l3. Mol. Phylogenet. Evol..

[25] Lai W.S., Wells M.L., Perera L., Blackshear P.J. (2019). The tandem zinc finger RNA binding domain of members of the tristetraprolin protein family. Wiley Interdiscip. Rev. RNA.

[26] Lai W.S., Perera L., Hicks S.N., Blackshear P.J. (2014). Mutational and structural analysis of the tandem zinc finger domain of tristetraprolin. J. Biol. Chem..

[27] Hudson B.P., Martinez-Yamout M.A., Dyson H.J., Wright P.E. (2004). Recognition of the mRNA AU-rich element by the zinc finger domain of TIS11d. Nat. Struct. Mol. Biol..

[28] Barreau C., Paillard L., Osborne H.B. (2005). AU-rich elements and associated factors: Are there unifying principles?. Nucleic Acids Res..

[29] Kovarik P., Bestehorn A., Fesselet J. (2021). Conceptual Advances in Control of Inflammation by the RNA-Binding Protein Tristetraprolin. Front. Immunol..

[30] Carreño A., Lykke-Andersen J. (2022). The Conserved CNOT1 Interaction Motif of Tristetraprolin Regulates ARE-mRNA Decay Independently of the p38 MAPK-MK2 Kinase Pathway. Mol. Cell Biol..

[31] Varnum B.C., Ma Q.F., Chi T.H., Fletcher B., Herschman H.R. (1991). The TIS11 primary response gene is a member of a gene family that encodes proteins with a highly conserved sequence containing an unusual Cys-His repeat. Mol. Cell Biol..

[32] Carballo E., Lai W.S., Blackshear P.J. (1998). Feedback inhibition of macrophage tumor necrosis factor-alpha production by tristetraprolin. Science.

[33] Zandhuis N.D., Guislain A., Popalzij A., Engels S., Popović B., Turner M., Wolkers M.C. (2024). Regulation of IFN-γ production by ZFP36L2 in T cells is time-dependent. Eur. J. Immunol..

[34] Taylor G.A., Carballo E., Lee D.M., Lai W.S., Thompson M.J., Patel D.D., Schenkman D.I., Gilkeson G.S., Broxmeyer H.E., Haynes B.F. (1996). A pathogenetic role for TNF alpha in the syndrome of cachexia, arthritis, and autoimmunity resulting from tristetraprolin (TTP) deficiency. Immunity.

[35] Maciej V.D., Mateva N., Schwarz J., Dittmers T., Mallick M., Urlaub H., Chakrabarti S. (2022). Intrinsically disordered regions of tristetraprolin and DCP2 directly interact to mediate decay of ARE-mRNA. Nucleic Acids Res..

[36] Stumpo D.J., Byrd N.A., Phillips R.S., Ghosh S., Maronpot R.R., Castranio T., Meyers E.N., Mishina Y., Blackshear P.J. (2004). Chorioallantoic fusion defects and embryonic lethality resulting from disruption of Zfp36L1, a gene encoding a CCCH tandem zinc finger protein of the Tristetraprolin family. Mol. Cell Biol..

[37] Bell S.E., Sanchez M.J., Spasic-Boskovic O., Santalucia T., Gambardella L., Burton G.J., Murphy J.J., Norton J.D., Clark A.R., Turner M. (2006). The RNA binding protein Zfp36l1 is required for normal vascularisation and post-transcriptionally regulates VEGF expression. Dev. Dyn..

[38] Redmon I.C., Ardizzone M., Hekimoğlu H., Hatfield B.M., Waldern J.M., Dey A., Montgomery S.A., Laederach A., Ramos S.B.V. (2022). Sequence and tissue targeting specificity of ZFP36L2 reveals Elavl2 as a novel target with co-regulation potential. Nucleic Acids Res..

[39] Ramos S.B., Stumpo D.J., Kennington E.A., Phillips R.S., Bock C.B., Ribeiro-Neto F., Blackshear P.J. (2004). The CCCH tandem zinc-finger protein Zfp36l2 is crucial for female fertility and early embryonic development. Development.

[40] Ball C.B., Rodriguez K.F., Stumpo D.J., Ribeiro-Neto F., Korach K.S., Blackshear P.J., Birnbaumer L., Ramos S.B.V. (2014). The RNA-Binding Protein, ZFP36L2, Influences Ovulation and Oocyte Maturation. PLoS ONE.

[41] Chousal J., Cho K., Ramaiah M., Skarbrevik D., Mora-Castilla S., Stumpo D.J., Lykke-Andersen J., Laurent L.C., Blackshear P.J., Wilkinson M.F. (2018). Chromatin Modification and Global Transcriptional Silencing in the Oocyte Mediated by the mRNA Decay Activator ZFP36L2. Dev. Cell.

[42] Noguchi A., Adachi S., Yokota N., Hatta T., Natsume T., Kawahara H. (2018). ZFP36L2 is a cell cycle-regulated CCCH protein necessary for DNA lesion-induced S-phase arrest. Biol. Open.

[43] Caulier G., Siblini J., Sène L., Mauxion F., Séraphin B. (2025). The CCR4-NOT complex: A multifaceted sensor of molecular signals instructing eukaryotic mRNA translation and stability. Nucleic Acids Res..

[44] Blake L.A., Watkins L., Liu Y., Inoue T., Wu B. (2024). A rapid inducible RNA decay system reveals fast mRNA decay in P-bodies. Nat. Commun..

[45] Tiedje C., Ronkina N., Tehrani M., Dhamija S., Laass K., Holtmann H., Kotlyarov A., Gaestel M. (2012). The p38/MK2-driven exchange between tristetraprolin and HuR regulates AU-rich element-dependent translation. PLoS Genet..

[46] Tao X., Gao G. (2015). Tristetraprolin Recruits Eukaryotic Initiation Factor 4E2 To Repress Translation of AU-Rich Element-Containing mRNAs. Mol. Cell. Biol..

[47] Fu R., Olsen M.T., Webb K., Bennett E.J., Lykke-Andersen J. (2016). Recruitment of the 4EHP-GYF2 cap-binding complex to tetraproline motifs of tristetraprolin promotes repression and degradation of mRNAs with AU-rich elements. RNA.

[48] Glauninger H., Wong Hickernell C.J., Bard J.A.M., Drummond D.A. (2022). Stressful steps: Progress and challenges in understanding stress-induced mRNA condensation and accumulation in stress granules. Mol. Cell.

[49] Kawai T., Ikegawa M., Ori D., Akira S. (2024). Decoding Toll-like receptors: Recent insights and perspectives in innate immunity. Immunity.

[50] Chen R., Zou J., Chen J., Zhong X., Kang R., Tang D. (2025). Pattern recognition receptors: Function, regulation and therapeutic potential. Signal Transduct. Target. Ther..

[51] Ma M., Jiang W., Zhou R. (2024). DAMPs and DAMP-sensing receptors in inflammation and diseases. Immunity.

[52] Mao H., Zhao X., Sun S.-c. (2025). NF-κB in inflammation and cancer. Cell. Mol. Immunol..

[53] Guilliams M., Ginhoux F., Jakubzick C., Naik S.H., Onai N., Schraml B.U., Segura E., Tussiwand R., Yona S. (2014). Dendritic cells, monocytes and macrophages: A unified nomenclature based on ontogeny. Nat. Rev. Immunol..

[54] Burn G.L., Foti A., Marsman G., Patel D.F., Zychlinsky A. (2021). The Neutrophil. Immunity.

[55] Sedlyarov V., Fallmann J., Ebner F., Huemer J., Sneezum L., Ivin M., Kreiner K., Tanzer A., Vogl C., Hofacker I. (2016). Tristetraprolin binding site atlas in the macrophage transcriptome reveals a switch for inflammation resolution. Mol. Syst. Biol..

[56] Kratochvill F., Machacek C., Vogl C., Ebner F., Sedlyarov V., Gruber A.R., Hartweger H., Vielnascher R., Karaghiosoff M., Rülicke T. (2011). Tristetraprolin-driven regulatory circuit controls quality and timing of mRNA decay in inflammation. Mol. Syst. Biol..

[57] Wang K.T., Wang H.H., Wu Y.Y., Su Y.L., Chiang P.Y., Lin N.Y., Wang S.C., Chang G.D., Chang C.J. (2015). Functional regulation of Zfp36l1 and Zfp36l2 in response to lipopolysaccharide in mouse RAW264.7 macrophages. J. Inflamm..

[58] Molle C., Zhang T., Ysebrant de Lendonck L., Gueydan C., Andrianne M., Sherer F., Van Simaeys G., Blackshear P.J., Leo O., Goriely S. (2013). Tristetraprolin regulation of interleukin 23 mRNA stability prevents a spontaneous inflammatory disease. J. Exp. Med..

[59] Ebner F., Sedlyarov V., Tasciyan S., Ivin M., Kratochvill F., Gratz N., Kenner L., Villunger A., Sixt M., Kovarik P. (2017). The RNA-binding protein tristetraprolin schedules apoptosis of pathogen-engaged neutrophils during bacterial infection. J. Clin. Investig..

[60] Vivier E., Artis D., Colonna M., Diefenbach A., Di Santo J.P., Eberl G., Koyasu S., Locksley R.M., McKenzie A.N.J., Mebius R.E. (2018). Innate Lymphoid Cells: 10 Years On. Cell.

[61] Nussbaum J.C., Van Dyken S.J., von Moltke J., Cheng L.E., Mohapatra A., Molofsky A.B., Thornton E.E., Krummel M.F., Chawla A., Liang H.E. (2013). Type 2 innate lymphoid cells control eosinophil homeostasis. Nature.

[62] Hikichi Y., Motomura Y., Takeuchi O., Moro K. (2021). Posttranscriptional regulation of ILC2 homeostatic function via tristetraprolin. J. Exp. Med..

[63] de Toeuf B., Melchior M., La C., Villanueva Alcantara A., Azouz A., Martens V., La C., Dubois I., Vande Velde S., Meyer L. (2025). Expansion of Interleukin-22-Producing Type 3 Innate Lymphoid Cells in the Gut of Tristetraprolin-Deficient Mice. Eur. J. Immunol..

[64] Hammad H., Lambrecht B.N. (2015). Barrier Epithelial Cells and the Control of Type 2 Immunity. Immunity.

[65] Niec R.E., Rudensky A.Y., Fuchs E. (2021). Inflammatory adaptation in barrier tissues. Cell.

[66] Bals R., Hiemstra P.S. (2004). Innate immunity in the lung: How epithelial cells fight against respiratory pathogens. Eur. Respir. J..

[67] Andrianne M., Assabban A., La C., Mogilenko D., Salle D.S., Fleury S., Doumont G., Van Simaeys G., Nedospasov S.A., Blackshear P.J. (2017). Tristetraprolin expression by keratinocytes controls local and systemic inflammation. JCI Insight.

[68] Assabban A., Dubois-Vedrenne I., Van Maele L., Salcedo R., Snyder B.L., Zhou L., Azouz A., de Toeuf B., Lapouge G., La C. (2021). Tristetraprolin expression by keratinocytes protects against skin carcinogenesis. JCI Insight.

[69] Bertesi M., Fantini S., Alecci C., Lotti R., Martello A., Parenti S., Carretta C., Marconi A., Grande A., Pincelli C. (2020). Promoter Methylation Leads to Decreased ZFP36 Expression and Deregulated NLRP3 Inflammasome Activation in Psoriatic Fibroblasts. Front. Med..

[70] Balakathiresan N.S., Bhattacharyya S., Gutti U., Long R.P., Jozwik C., Huang W., Srivastava M., Pollard H.B., Biswas R. (2009). Tristetraprolin regulates IL-8 mRNA stability in cystic fibrosis lung epithelial cells. Am. J. Physiol.-Lung Cell. Mol. Physiol..

[71] Rynne J., Ortiz-Zapater E., Bagley D.C., Zanin O., Doherty G., Kanabar V., Ward J., Jackson D.J., Parsons M., Rosenblatt J. (2023). The RNA binding proteins ZFP36L1 and ZFP36L2 are dysregulated in airway epithelium in human and a murine model of asthma. Front. Cell Dev. Biol..

[72] Eshelman M.A., Matthews S.M., Schleicher E.M., Fleeman R.M., Kawasawa Y.I., Stumpo D.J., Blackshear P.J., Koltun W.A., Ishmael F.T., Yochum G.S. (2019). Tristetraprolin targets Nos2 expression in the colonic epithelium. Sci. Rep..

[73] Moore M.J., Blachere N.E., Fak J.J., Park C.Y., Sawicka K., Parveen S., Zucker-Scharff I., Moltedo B., Rudensky A.Y., Darnell R.B. (2018). ZFP36 RNA-binding proteins restrain T cell activation and anti-viral immunity. eLife.

[74] Petkau G., Mitchell T.J., Chakraborty K., Bell S.E., D’Angeli V., Matheson L., Turner D.J., Saveliev A., Gizlenci O., Salerno F. (2022). The timing of differentiation and potency of CD8 effector function is set by RNA binding proteins. Nat. Commun..

[75] Petkau G., Mitchell T.J., Evans M.J., Matheson L., Salerno F., Turner M. (2024). Zfp36l1 establishes the high-affinity CD8 T-cell response by directly linking TCR affinity to cytokine sensing. Eur. J. Immunol..

[76] Zandhuis N.D., Bradarić A., van der Zwaan C., Hoogendijk A.J., Popović B., Wolkers M.C. (2025). Combined Deletion of ZFP36L1 and ZFP36L2 Drives Superior Cytokine Production in T Cells at the Cost of Cell Fitness. Eur. J. Immunol..

[77] Makita S., Takatori H., Iwata A., Tanaka S., Furuta S., Ikeda K., Suto A., Suzuki K., Ramos S.B.V., Nakajima H. (2020). RNA-Binding Protein ZFP36L2 Downregulates Helios Expression and Suppresses the Function of Regulatory T Cells. Front. Immunol..

[78] Newman R., Ahlfors H., Saveliev A., Galloway A., Hodson D.J., Williams R., Besra G.S., Cook C.N., Cunningham A.F., Bell S.E. (2017). Maintenance of the marginal-zone B cell compartment specifically requires the RNA-binding protein ZFP36L1. Nat. Immunol..

[79] Saveliev A., Bell S.E., Turner M. (2021). Efficient homing of antibody-secreting cells to the bone marrow requires RNA-binding protein ZFP36L1. J. Exp. Med..

[80] Salerno F., Whale A.J., Matheson L.S., Vespasiani D., Foster W.S., Mitchell T.J., Screen M., Stammers M., Bell S.E., Hodson D.J. (2025). RNA binding proteins control the G_2_-M checkpoint of the germinal center B cell. Sci. Immunol..

[81] Lin R.J., Huang C.H., Liu P.C., Lin I.C., Huang Y.L., Chen A.Y., Chiu H.P., Shih S.R., Lin L.H., Lien S.P. (2020). Zinc finger protein ZFP36L1 inhibits influenza A virus through translational repression by targeting HA, M and NS RNA transcripts. Nucleic Acids Res..

[82] Chiu H., Chiu H.P., Yu H.P., Lin L.H., Chen Z.P., Lin Y.L., Lin R.J. (2022). Zinc Finger Protein ZFP36L1 Inhibits Flavivirus Infection by both 5′-3′ XRN1 and 3′-5′ RNA-Exosome RNA Decay Pathways. J. Virol..

[83] Wang Z., Hou P., Wu Y., Dai J., Zhao P., Cheng X., Hu Z., Zhang L., Hua J. (2025). ZFP36 reverses chemoresistance by disrupting lipid droplets accumulation in non-small cell lung cancer. Cell. Signal..

[84] Chen J., Patial S., Saini Y. (2022). Silencing of RNA binding protein, ZFP36L1, promotes epithelial-mesenchymal transition in liver cancer cells by regulating transcription factor ZEB2. Cell. Signal..

[85] Wei X., Liu J., Cheng J., Cai W., Xie W., Wang K., Lin L., Hou J., Cai J., Zhuo H. (2024). Super-enhancer-driven ZFP36L1 promotes PD-L1 expression in infiltrative gastric cancer. eLife.

[86] Yuan S., Zhai Y., Tao T., Zhang X., Bashir G., Li G., Wang G., Wu S. (2022). Conflicting Roles of ZFP36L1 in Regulating the Progression of Muscle Invasive Bladder Cancer. Front. Mol. Biosci..

[87] Yonemori K., Seki N., Kurahara H., Osako Y., Idichi T., Arai T., Koshizuka K., Kita Y., Maemura K., Natsugoe S. (2017). ZFP36L2 promotes cancer cell aggressiveness and is regulated by antitumor microRNA-375 in pancreatic ductal adenocarcinoma. Cancer Sci..

[88] Zhang Y., Tian F., Zhao J. (2023). MiR-520d-3p suppresses the proliferation and epithelial-mesenchymal transition of cervical cancer cells by targeting ZFP36L2. Heliyon.

[89] Wan L., Jia Y., Chen N., Zheng S. (2024). Circ_0003789 Knockdown Inhibits Tumor Progression by miR-429/ZFP36L2 Axis in Gastric Cancer. Biochem. Genet..

[90] Zhu T., Shen Q., Shen L., Wang Y., Zhu B., Ma L., Feng S., Wang C., Yan S., Li J. (2025). Senescence-induced p21^high^ macrophages contributed to CD8^+^ T cells-related immune hyporesponsiveness in kidney transplantation via Zfp36/IL-27 axis. Cell Discov..

[91] Mai D., Boyce T., Mehta A., Reff J., Scholler J., Sheppard N.C., June C.H. (2024). ZFP36 disruption is insufficient to enhance the function of mesothelin-targeting human CAR-T cells. Sci. Rep..

[92] Wilfahrt D., Delgoffe G.M. (2024). Metabolic waypoints during T cell differentiation. Nat. Immunol..

[93] Giles J.R., Globig A.M., Kaech S.M., Wherry E.J. (2023). CD8^+^ T cells in the cancer-immunity cycle. Immunity.

[94] Choi J.O., Ham J.H., Hwang S.S. (2022). RNA Metabolism in T Lymphocytes. Immune Netw..

[95] Popović B., Nicolet B.P., Guislain A., Engels S., Jurgens A.P., Paravinja N., Freen-van Heeren J.J., van Alphen F.P.J., van den Biggelaar M., Salerno F. (2023). Time-dependent regulation of cytokine production by RNA binding proteins defines T cell effector function. Cell Rep..

[96] Wang L., Liang Y., Zhao C., Ma P., Zeng S., Ju D., Zhao M., Yu M., Shi Y. (2025). Regulatory T cells in homeostasis and disease: Molecular mechanisms and therapeutic potential. Signal Transduct. Target. Ther..

[97] Contreras-Castillo E., García-Rasilla V.Y., García-Patiño M.G., Licona-Limón P. (2024). Stability and plasticity of regulatory T cells in health and disease. J. Leukoc. Biol..

[98] Sakaguchi S., Yamaguchi T., Nomura T., Ono M. (2008). Regulatory T cells and immune tolerance. Cell.

[99] Liu X., Zhang W., Han Y., Cheng H., Liu Q., Ke S., Zhu F., Lu Y., Dai X., Wang C. (2024). FOXP3^+^ regulatory T cell perturbation mediated by the IFNγ-STAT1-IFITM3 feedback loop is essential for anti-tumor immunity. Nat. Commun..

[100] Wing J.B., Ise W., Kurosaki T., Sakaguchi S. (2014). Regulatory T cells control antigen-specific expansion of Tfh cell number and humoral immune responses via the coreceptor CTLA-4. Immunity.

[101] Sage P.T., Paterson A.M., Lovitch S.B., Sharpe A.H. (2014). The coinhibitory receptor CTLA-4 controls B cell responses by modulating T follicular helper, T follicular regulatory, and T regulatory cells. Immunity.

[102] Permanyer M., Bošnjak B., Glage S., Friedrichsen M., Floess S., Huehn J., Patzer G.E., Odak I., Eckert N., Zargari R. (2021). Efficient IL-2R signaling differentially affects the stability, function, and composition of the regulatory T-cell pool. Cell Mol. Immunol..

[103] Cyster J.G., Allen C.D.C. (2019). B Cell Responses: Cell Interaction Dynamics and Decisions. Cell.

[104] Victora G.D., Nussenzweig M.C. (2022). Germinal Centers. Annu. Rev. Immunol..

[105] Broketa M., Bruhns P. (2021). Single-Cell Technologies for the Study of Antibody-Secreting Cells. Front. Immunol..

[106] Kaech S.M., Cui W. (2012). Transcriptional control of effector and memory CD8+ T cell differentiation. Nat. Rev. Immunol..

[107] McLane L.M., Abdel-Hakeem M.S., Wherry E.J. (2019). CD8 T Cell Exhaustion During Chronic Viral Infection and Cancer. Annu. Rev. Immunol..

[108] Saini Y., Chen J., Patial S. (2020). The Tristetraprolin Family of RNA-Binding Proteins in Cancer: Progress and Future Prospects. Cancers.

[109] Zhang D., Zhou Z., Yang R., Zhang S., Zhang B., Tan Y., Chen L., Li T., Tu J. (2021). Tristetraprolin, a Potential Safeguard Against Carcinoma: Role in the Tumor Microenvironment. Front. Oncol..

[110] Sobolewski C., Dubuquoy L., Legrand N. (2022). MicroRNAs, Tristetraprolin Family Members and HuR: A Complex Interplay Controlling Cancer-Related Processes. Cancers.

[111] Mao Y., Lv M., Cao W., Liu X., Cui J., Wang Y., Wang Y., Nie G., Liu X., Wang H. (2020). Circular RNA 000554 represses epithelial-mesenchymal transition in breast cancer by regulating microRNA-182/ZFP36 axis. FASEB J..

[112] Brennan S.E., Kuwano Y., Alkharouf N., Blackshear P.J., Gorospe M., Wilson G.M. (2009). The mRNA-destabilizing protein tristetraprolin is suppressed in many cancers, altering tumorigenic phenotypes and patient prognosis. Cancer Res..

[113] Liang J., Lei T., Song Y., Yanes N., Qi Y., Fu M. (2009). RNA-destabilizing factor tristetraprolin negatively regulates NF-kappaB signaling. J. Biol. Chem..

[114] Schichl Y.M., Resch U., Hofer-Warbinek R., de Martin R. (2009). Tristetraprolin impairs NF-kappaB/p65 nuclear translocation. J. Biol. Chem..

[115] Hubiernatorova A., Novak J., Vaskovicova M., Sekac D., Kropyvko S., Hodny Z. (2025). Tristetraprolin affects invasion-associated genes expression and cell motility in triple-negative breast cancer model. Cytoskeleton.

[116] Lee W.H., Lee H.H., Vo M.-T., Kim H.J., Ko M.S., Im Y.-C., Min Y.J., Lee B.J., Cho W.J., Park J.W. (2011). Casein Kinase 2 Regulates the mRNA-destabilizing Activity of Tristetraprolin. J. Biol. Chem..

[117] Lee H.H., Son Y.J., Lee W.H., Park Y.W., Chae S.W., Cho W.J., Kim Y.M., Choi H.J., Choi D.H., Jung S.W. (2010). Tristetraprolin regulates expression of VEGF and tumorigenesis in human colon cancer. Int. J. Cancer.

[118] Fudhaili A., Yoon N.A., Kang S., Ryu J., Jeong J.Y., Lee D.H., Kang S.S. (2019). Resveratrol epigenetically regulates the expression of zinc finger protein 36 in non-small cell lung cancer cell lines. Oncol. Rep..

[119] Vogel K.U., Bell L.S., Galloway A., Ahlfors H., Turner M. (2016). The RNA-Binding Proteins Zfp36l1 and Zfp36l2 Enforce the Thymic β-Selection Checkpoint by Limiting DNA Damage Response Signaling and Cell Cycle Progression. J. Immunol..

[120] Deka K., Carter J.M., Bahai A., Ang D.A., Sim N., Chong H.Y., Lee G.H.B., Tan S.M., Chng W.J., Kappei D. (2025). Multiple myeloma associated long non-coding RNA PLUM confers chemoresistance by enhancing PRC2 mediated UPR pathway activation. Nat. Commun..

[121] Wang Q., Ning H., Peng H., Wei L., Hou R., Hoft D.F., Liu J. (2017). Tristetraprolin inhibits macrophage IL-27-induced activation of antitumour cytotoxic T cell responses. Nat. Commun..

[122] Ding K., Zhang F., Qi G., Lin M., Chen M., Chen Y., Zheng J., Zhou F. (2023). ZFP36L1 Promotes Gastric Cancer Progression via Regulating JNK and p38 MAPK Signaling Pathways. Recent Pat. Anticancer Drug Discov..

[123] Zhou M., Li J., Chen C. (2022). High expression of ZFP36L2 correlates with the prognosis and immune infiltration in lower-grade glioma. Front. Genet..

[124] Dolicka D., Sobolewski C., Gjorgjieva M., Correia de Sousa M., Berthou F., De Vito C., Colin D.J., Bejuy O., Fournier M., Maeder C. (2021). Tristetraprolin Promotes Hepatic Inflammation and Tumor Initiation but Restrains Cancer Progression to Malignancy. Cell. Mol. Gastroenterol. Hepatol..

[125] Xie S., Wu H., Li X., Wang B., Liu Y., Li C., Huang Q., Yang Y., Gu S. (2026). A comprehensive cancer analysis investigating the oncogenic role of zinc finger protein 36 (ZFP36) in human tumors. Sci. Rep..

[126] Pan Q.H., Fan Y.H., Wang Y.Z., Li D.M., Hu C.E., Li R.X. (2020). Long noncoding RNA NNT-AS1 functions as an oncogene in breast cancer via repressing ZFP36 expression. J. Biol. Regul. Homeost. Agents.

[127] Smallie T., Ross E.A., Ammit A.J., Cunliffe H.E., Tang T., Rosner D.R., Ridley M.L., Buckley C.D., Saklatvala J., Dean J.L. (2015). Dual-Specificity Phosphatase 1 and Tristetraprolin Cooperate To Regulate Macrophage Responses to Lipopolysaccharide. J. Immunol..

[128] Ross E.A., Naylor A.J., O’Neil J.D., Crowley T., Ridley M.L., Crowe J., Smallie T., Tang T.J., Turner J.D., Norling L.V. (2017). Treatment of inflammatory arthritis via targeting of tristetraprolin, a master regulator of pro-inflammatory gene expression. Ann. Rheum. Dis..

[129] Uehara Y., Suzukawa M., Horie M., Igarashi S., Minegishi M., Takada K., Saito A., Nagase H. (2024). ZFP36 family expression is suppressed by Th2 cells in asthma, leading to enhanced synthesis of inflammatory cytokines and cell surface molecules. Cell Immunol..

[130] Xing D., Cao H., Yang Y., Liu S., Yu H., Liu Z., Wang K., Wei X., Yan A. (2026). RNA-binding protein tristetraprolin inhibits Th2 cell activation and differentiation in allergic rhinitis by promoting TRIM18 mRNA decay. J. Biol. Chem..

[131] Zhao W., Liu M., D’Silva N.J., Kirkwood K.L. (2011). Tristetraprolin regulates interleukin-6 expression through p38 MAPK-dependent affinity changes with mRNA 3′ untranslated region. J. Interferon Cytokine Res..

[132] D’Mello C., Swain M.G. (2017). Immune-to-Brain Communication Pathways in Inflammation-Associated Sickness and Depression. Curr. Top. Behav. Neurosci..

[133] Heneka M.T., Carson M.J., El Khoury J., Landreth G.E., Brosseron F., Feinstein D.L., Jacobs A.H., Wyss-Coray T., Vitorica J., Ransohoff R.M. (2015). Neuroinflammation in Alzheimer’s disease. Lancet Neurol..

[134] Sweeney M.D., Zhao Z., Montagne A., Nelson A.R., Zlokovic B.V. (2019). Blood-Brain Barrier: From Physiology to Disease and Back. Physiol. Rev..

[135] Liu T., Chen D., Liu F., Sun Y. (2026). ZFP36-mediated ZBP1 degradation inhibits microglia pro-inflammatory and NLRP3 inflammasome activation in Alzheimer’s disease. Cell Biol. Toxicol..

[136] Clark A.R., Dean J.L. (2016). The control of inflammation via the phosphorylation and dephosphorylation of tristetraprolin: A tale of two phosphatases. Biochem. Soc. Trans..

[137] Li S., Liu S., Chen R.A., Huang M., Fung T.S., Liu D.X. (2022). Activation of the MKK3-p38-MK2-ZFP36 Axis by Coronavirus Infection Restricts the Upregulation of AU-Rich Element-Containing Transcripts in Proinflammatory Responses. J. Virol..

[138] Zanfi E.D., Fantini S., Lotti R., Bertesi M., Marconi A., Grande A., Manfredini R., Pincelli C., Zanocco-Marani T. (2020). Wnt/CTNNB1 Signal Transduction Pathway Inhibits the Expression of ZFP36 in Squamous Cell Carcinoma, by Inducing Transcriptional Repressors SNAI1, SLUG and TWIST. Int. J. Mol. Sci..

[139] Tréguer K., Faucheux C., Veschambre P., Fédou S., Thézé N., Thiébaud P. (2013). Comparative functional analysis of ZFP36 genes during Xenopus development. PLoS ONE.

[140] Bennett C.F., Krainer A.R., Cleveland D.W. (2019). Antisense Oligonucleotide Therapies for Neurodegenerative Diseases. Annu. Rev. Neurosci..

[141] Patial S., Blackshear P.J. (2016). Tristetraprolin as a Therapeutic Target in Inflammatory Disease. Trends Pharmacol. Sci..

[142] Tiedje C., Diaz-Muñoz M.D., Trulley P., Ahlfors H., Laaß K., Blackshear P.J., Turner M., Gaestel M. (2016). The RNA-binding protein TTP is a global post-transcriptional regulator of feedback control in inflammation. Nucleic Acids Res..

[143] Mukherjee N., Jacobs N.C., Hafner M., Kennington E.A., Nusbaum J.D., Tuschl T., Blackshear P.J., Ohler U. (2014). Global target mRNA specification and regulation by the RNA-binding protein ZFP36. Genome Biol..

